# Complexity Analysis of Skin Nerve Activity for Quantitative Assessment of Acute Sympathetic Nervous System Activation

**DOI:** 10.3390/s26051611

**Published:** 2026-03-04

**Authors:** Youngsun Kong, Yubin Choi, Farnoush Baghestani, Dong-Guk Shin, I-Ping Chen, Ki Chon

**Affiliations:** 1 Department of Biomedical Engineering, University of Connecticut, Storrs, CT 06269, USA; farnoush.baghestani@uconn.edu (F.B.); ki.chon@uconn.edu (K.C.); 2 Department of Endodontics, University of Connecticut Health, Farmington, CT 06032, USA; yuchoi@uchc.edu (Y.C.); ipchen@uchc.edu (I.-P.C.); 3 Department of Computer Science and Engineering, University of Connecticut, Storrs, CT 06269, USA; dong.shin@uconn.edu

**Keywords:** skin sympathetic nerve activity, complexity analysis, entropy, Hjorth parameters, fractal dimension, autonomic nervous system, dental pain, acute sympathetic activation

## Abstract

Skin nerve activity (SKNA), extracted from electrocardiograms, is a noninvasive surrogate of sympathetic nervous system (SNS) activity. We evaluated whether complexity-based metrics derived from integrated SKNA (iSKNA; 500–1000 Hz) and time-varying SKNA (TVSKNA; 160–1140 Hz) discriminate SNS activation in experimental (*n* = 23) and clinical dental datasets (*n* = 49). Experimental tasks included the Valsalva maneuver and thermal grill stimulation; clinical recordings involved cold testing, with exploratory subgroup analyses based on anxiety status. Pain intensity was assessed using a visual analog scale (VAS); clinically significant pain (CSP+) was defined as a VAS score ≥ 4. Approximate entropy, sample entropy, Hjorth mobility and complexity, Katz fractal dimension, and standard deviation were computed. In the experimental dataset, the Valsalva maneuver produced large-to-huge effects (Cohen’s *d* = 1.93–3.46, *p* < 0.001). Thermal grill tasks showed moderate-to-large effects for adjacent pain levels (|*d*| = 0.63–0.71). ROC analysis showed strong discrimination for baseline vs. pain (AUC 0.80–0.99) but limited separation between adjacent pain levels (AUC 0.56–0.64). In the clinical dataset, discrimination was strongest for no pain vs. CSP+ (|*d*| = 0.86–1.17), with higher AUC in severe-anxiety participants (0.81–0.96) than non-severe (0.64–0.75). Complexity measures generally decreased during SNS activation, complementing amplitude-based changes. These findings support combined magnitude- and complexity-based descriptors for characterizing short-term sympathetic activation.

## 1. Introduction

Skin nerve activity (SKNA), derived from electrocardiogram (ECG) signals recorded at high sampling frequencies (≥2 kHz), has recently emerged as a novel, non-invasive surrogate for assessing sympathetic nervous system (SNS) activity. It offers faster response time compared to traditional SNS-assessing methods [1,2,3,4]. For example, heart rate variability (HRV) requires longer time windows to reliably capture variability in cardiac beat intervals (typically ≥ 10 s) [5], and electrodermal activity depends on sweat gland physiology, which involves additional glandular and neurotransmitter processes that introduce delays relative to direct nerve firings [6]. Additionally, previous studies have demonstrated superior discriminative ability using SKNA over these modalities [1,2,3,4].

Kusayama et al. first identified electrical signals associated with skin sympathetic nerve activity (SSNA) in ECG recordings and termed them SKNA to differentiate them from SSNA, which is measured invasively using microneurography [7]. They also introduced a method for extracting SKNA from ECG recordings, known as neuECG, which applies a bandpass filter with cutoff frequencies between 0.5 and 1 kHz, followed by rectification and a moving average filter to derive integrated SKNA (iSKNA) [7,8]. Attenuation of iSKNA following stellate ganglion blockade suggested that SKNA primarily originates from the stellate ganglion, a key structure in the SNS [9].

SKNA is typically assessed through iSKNA via the neuECG technique. More recently, a time-varying approach known as TVSKNA has been introduced, demonstrating greater sensitivity, reproducibility, and accuracy in detecting SNS activity [10]. Both iSKNA and TVSKNA have primarily been analyzed using simple descriptive statistics, such as the mean and standard deviation. For example, elevated mean and standard deviation values of iSKNA have been observed during cold pressor testing [7] and ventricular arrhythmia [11]. Additionally, recent studies have reported increases in the maximum, mean, and standard deviation of both iSKNA and TVSKNA during the Valsalva maneuver (VM), Stroop test, and pain stimulation tasks [1,10].

To the best of our knowledge, prior analyses of SKNA have relied primarily on descriptive measures, such as the mean and standard deviation, and burst counts and rates. These metrics quantify overall signal magnitude and variability and have been useful in characterizing sympathetic activity. However, they do not explicitly capture temporal ordering, irregularity, or structural organization of SKNA bursts. In this study, temporal organization refers to the sequential structure of samples within each analysis segment rather than time-resolved tracking across windows. SKNA consists of intermittent postganglionic sympathetic discharges characterized by transient bursts and time-varying amplitude modulation, suggesting non-stationary temporal behavior. Complexity-based measures, including entropy and fractal metrics, provide quantitative descriptors of temporal irregularity and structural organization and have been successfully applied to other neurophysiological signals such as HRV, EEG, and fMRI [12,13,14,15,16]. Therefore, applying these measures to SKNA may offer complementary characterization of sympathetic dynamics beyond amplitude variability alone.

Accordingly, the primary objective of this study was to evaluate whether complexity-based measures of SKNA can detect short-term SNS activation in both experimental and clinical pain settings. The secondary objective was to assess the discriminative ability of these measures to differentiate across physiological states, including baseline versus SNS activation and clinically defined pain conditions, quantified using effect sizes and area under (AUC) the receiver operating characteristic (ROC) curve. We hypothesized that complexity-based SKNA features would differ across these conditions and provide meaningful discriminative performance.

## 2. Materials and Methods

### 2.1. Study Design Overview

This study consisted of two complementary datasets: an experimental dataset involving 24 healthy participants undergoing VM and thermal grill (TG) pain tasks, and a clinical dataset involving 49 dental patients undergoing cold testing. ECG signals were recorded at a sampling frequency of 10 kHz in both settings. SKNA was extracted from ECG recordings using time-series processing methods that generated both integrated (iSKNA) and time-varying SKNA (TVSKNA) representations (described in detail in Section 2.3).

**Figure 1:**
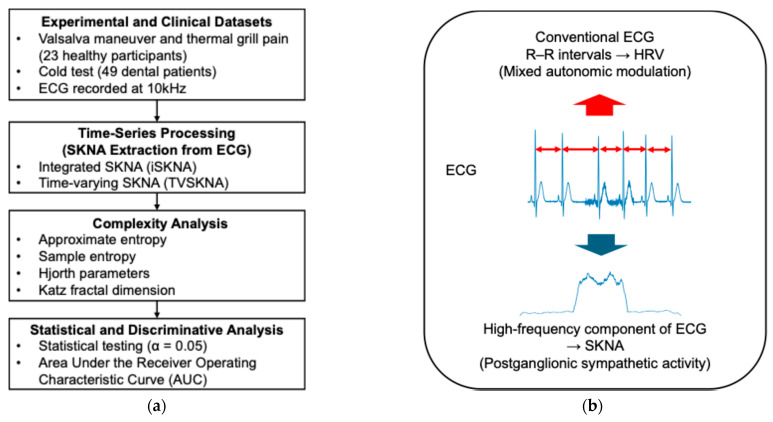
Overview of the study design and analytical pipeline (**a**) and schematic distinction between ECG-derived HRV and high-frequency SKNA (**b**). ECG: electrocardiogram. SKNA: skin nerve activity.

Complexity features—including entropy measures, Hjorth parameters, and the Katz fractal dimension (KFD)—were subsequently computed from the extracted SKNA signals. Statistical comparisons and discriminative performance analyses were then conducted to evaluate differentiation across physiological conditions. The overall workflow is illustrated in Figure 1.

For clarity, HRV is derived from beat-to-beat R–R interval variability within the conventional ECG bandwidth and reflects combined sympathetic and parasympathetic modulation of cardiac rhythm. In contrast, SKNA reflects postganglionic sympathetic nerve discharges extracted from the high-frequency component of surface ECG recordings.

### 2.2. Datasets

We collected two datasets: (1) an experimental dataset from healthy participants who underwent the VM and TG pain, and (2) a clinical dataset of cold testing using endo-ice refrigerant spray (Coltene, Altstätten, Switzerland) in the Endodontic Clinic at UConn Health. All human studies were in accordance with the guidelines of the Institutional Review Board of the University of Connecticut and University of Connecticut Health (IRB protocol H21-0155 and H24-0203 for experimental dataset; IRB protocol 20-043-1 for clinical study).

#### 2.2.1. Experimental Dataset

##### Participants

Twenty-four participants (13 males and 11 females) aged between 20 and 57 underwent VM and TG testing in a random order, during which ECG signals were recorded.

##### Valsalva Maneuver (VM)

VM was performed by forcefully exhaling against a closed airway, a procedure commonly used to assess autonomic nervous system function [17,18]. Participants were instructed to perform a deep inhalation followed by sustained forceful exhalation for up to 60 s. However, because some participants were unable to maintain the maneuver for the full duration, a minimum sustained strain period of 20 s was required for inclusion in the analysis to ensure consistent segment length across participants. The procedure was repeated three to six times per participant. Pressure was not directly monitored using a manometer; participants were verbally instructed and coached to maintain a consistent effort throughout the maneuver.

##### Thermal Grill Illusion

TG illusion is a sensory illusion where interlaced cold and warm stimuli applied to the skin evoke a burning heat sensation without causing tissue injury [19]. Studies have demonstrated that TG stimulation induces varying levels of acute pain, increases blood pressure, heart rate [20], and electrodermal activity [21], indicating increased SNS activity.

In our experiment, three types of TGs were used to induce varying levels of pain intensities: TG_low_, TG_high_, and TG_sham_. Cold pipes were supplied using an ice-water bath. Warm water temperature was individually adjusted within a range of approximately 45–60 °C to induce pain levels of 4–6 on a 10-point visual analog scale (VAS) for TG_low_ and VAS ≥ 7 for TG_high_. Because of heat loss and transmission characteristics of the grill apparatus, the exact surface temperature at the time of contact was not directly recorded. Pain intensity was therefore calibrated based on participant-reported VAS ratings rather than absolute temperature.

Participants placed only their left hands on the TGs for consistency and practical feasibility. To minimize the effect of pain anticipation, the participants wore blindfolds, and the TGs were placed on a wheeled table. To accurately place the participants’ hands on the grills, two verbal cues were used: ‘ready’ and ‘go’. Upon hearing ‘ready’, participants place their left hand slightly to their left. Then, an experimenter adjusted the position of the wheeled table to locate the target grill directly below the hand. Upon hearing ‘go’, participants lowered their left hand onto the grill. Contact duration was approximately 5 s or until voluntary withdrawal if the stimulus became intolerable. The participants then reported their pain levels on a 0–10 VAS. Three stimuli were given for each grill, for a total of nine stimuli, in a randomized order. The interstimulus interval was approximately 40 s.

TG_sham_ (cold-only pipes) was excluded from analysis because cold stimulation of the hand can independently evoke sympathetic activation and does not represent a physiologically neutral control condition.

##### Electrocardiogram and Skin Nerve Activity

ECG signals were recorded using a BioAmp with a PowerLab device (ADInstrument, Sydney, Australia). ECG signals were collected using three Ag/AgCl electrodes with two different configurations. For sixteen participants, two electrodes were placed on the inner sides of both wrists. For the other eight participants, one electrode was placed on either the left or right wrist (randomly assigned), and the other on the inner side of the contralateral ankle. For all participants, a reference electrode was attached to the left ribcage area. Detailed ECG acquisition settings are described in Section 2.2.3. SKNA was extracted from the ECG recordings (Please see Section 2.3).

##### Design and Procedure

Participants were asked to refrain from consuming any caffeine-containing or stimulating substances for 24 h before their experiment day. The experiments took place in a 10 × 10 m lab located in the Engineering and Science building at the University of Connecticut’s Storrs campus. Before each experiment, participants completed a medical screening questionnaire and a consent form. After cleaning the skin with 70% isopropyl alcohol, Ag/AgCl electrodes were attached to the participants for ECG recording. Participants then underwent VM and TG in a random order. Prior to each task, a 2 min baseline recording was conducted. For analysis, fixed-length baseline segments were extracted from the middle portion of the baseline recording to avoid transitional effects. (e.g., for 10 s analyses, segments were extracted from 55 to 65 s of the baseline recording). Baseline segments served as the control condition for VM and as the no-pain reference condition for TG in statistical and discriminative analyses.

For TG, three pain groups were analyzed in this study: (1) baseline, representing no pain (VAS = 0); (2) clinically non-significant pain, defined as (0 < VAS < 4); and (3) clinically significant pain, defined as VAS ≥ 4. Groups 1, 2, and 3 were labeled as NP, CSP−, and CSP+, respectively. This pain threshold score is commonly used to distinguish between clinically non-significant and significant pain [22,23,24,25].

Although TG stimulus intensities were predefined to target moderate (VAS 4–6; TG_low_) and high (VAS ≥ 7; T_Ghigh_) pain levels for experimental control, actual reported VAS ratings may vary due to individual differences and mild adaptation during repeated stimulation. Therefore, the final statistical grouping was based solely on reported VAS scores rather than stimulus targets. Pain was categorized using the clinically established threshold (VAS ≥ 4) to define clinically significant pain (CSP+) and VAS < 4 to define CSP−. This approach ensured that pain categorization reflected actual subjective intensity and maintained consistency with the clinical cold-test dataset (see Section 2.2.2), where the same clinical criterion was applied. Note that the sham group was excluded from analysis due to uncertainty regarding whether the increases in SKNA were attributed to stress or cold stimulus.

#### 2.2.2. Clinical Dataset

##### Participants

Fifty patients (21 males and 29 females) whose ages were between 21 and 68 were recruited for our study. Participants had at least one tooth requiring root canal treatment and two nearby healthy teeth. We excluded patients under the age of 18, pregnant women, those with porcelain-crowned teeth, individuals taking medications with anticholinergic side effects, which can affect skin conductance, those with a prior history of sympathectomy procedures, and patients diagnosed with Raynaud’s syndrome.

##### Cold Testing

Cold testing uses a refrigerant spray Endo-ice, which can reach a temperature as low as −26.2 °C, to differentiate between normal pulp, pulpal necrosis, and pulpitis [26]. This stimulates Aδ nerve fibers by causing an outward flow of dentinal tubular fluid [27]. In addition, increased electrodermal activity has been observed [21]. Mild and intense sensations are expected for normal and pulpitis teeth, respectively, whereas no response is typically observed with necrotic teeth [28].

In our study, a dentist applied Endo-ice to a cotton pellet and placed it on the surface of the tested teeth. The duration of application was not formally timed but was brief and consistent with routine clinical cold testing procedures. A sham test was also conducted by spraying into the air—allowing patients to hear the sound—while a clean, unsprayed cotton pellet was placed on the tooth to control for auditory and tactile cues. Sham trials were not analyzed as a separate condition; instead, all stimuli were categorized according to the predefined VAS-based pain-response criteria described in Section Design and Procedure below.

Multiple teeth per patient were included when clinically indicated. The interstimulus interval was not strictly timed but was typically greater than approximately 20 s to allow perceptual recovery between tests. Because recordings were obtained in a routine clinical environment, a strictly controlled baseline recording prior to each individual tooth test was not consistently available.

After each stimulus, patients reported their pain levels on a 0–10 VAS. For the present analysis, teeth diagnosed as healthy, pulpitis, or necrotic were pooled and categorized according to pain-response groups (see Section Design and Procedure for details).

##### Corah’s Dental Anxiety Scale (DAS)

The DAS consists of a four-question survey with each question offering five response options. The total score ranges from 4 to 20, with the following interpretations: moderate (scores 9–12), high anxiety (scores 13–14), and severe anxiety (scores 15–20) [29]. We defined the severe anxiety (SA) group as patients with total scores of 15 or higher, and the non-severe anxiety (NSA) group as those with scores below 15.

Anxiety level was used as an exploratory stratification variable in secondary ROC analyses to assess whether discriminative performance differed by anxiety status. Mixed-effects modeling did not include anxiety as a primary factor. Statistical analyses for AUC comparisons were conducted separately within severe-anxiety (SA) and non-severe-anxiety (NSA) groups. The distribution of SA and NSA participants is summarized in Section 2.2.4 and Table 1.

##### Electrocardiogram and Skin Nerve Activity

ECG signals were recorded using the same device as the experimental dataset. The signals were collected using Ag/AgCl electrodes placed on the inner wrist of the non-dominant hand and the inner ankle on the contralateral side. A reference electrode was attached to the inner ankles on the same side as the dominant hand. Detailed ECG acquisition settings are described in Section 2.2.3. SKNA was subsequently extracted from the ECG recordings (Please see Section 2.3).

##### Design and Procedure

The experiments took place in a dental clinic at the University of Connecticut Health in Farmington, CT. Before each experiment, participants completed informed consent, Health Insurance Portability and Accountability Act (HIPAA) authorization forms, and DAS. After cleaning the skin using 70% isopropyl alcohol wipes, Ag/AgCl electrodes were attached for ECG recording. Participants then underwent the cold test following a minimum of two minutes of seated relaxation to allow physiological stabilization. Due to the variability inherent in the routine clinical setting, this relaxation period was not consistently used as a formal analytical baseline.

Using the same threshold criteria as in the TG pain analysis, three pain groups were established for this dataset: (1) no pain (VAS = 0), (2) clinically non-significant pain (0 < VAS < 4), and (3) clinically significant pain (VAS ≥ 4), labeled as NP, CSP−, and CSP+, respectively. Note that the definition of pain in our analyses was based solely on the VAS, whereas clinically, dental pain typically refers to an intense and/or lingering sensation resulting from pulpitis.

#### 2.2.3. Electrocardiogram Acquisition Settings

ECG signals were recorded at a sampling frequency of 10 kHz with the acquisition input range set to 5 mV, consistent with previously published neuECG methodologies [7]. Data were digitized using the PowerLab analog-to-digital converter with 16-bit resolution (ADInstruments, Sydney, Australia). No additional analog bandpass filtering was applied during acquisition; broadband signals were recorded to preserve high-frequency components relevant to SKNA extraction. Ag/AgCl surface electrodes were used for all recordings, and skin was cleaned with alcohol prior to electrode placement to reduce impedance and improve signal quality. All bandpass and highpass filtering procedures were performed offline during preprocessing after SKNA extraction.

#### 2.2.4. Final Sample and Unit of Analysis

After preprocessing and exclusion of segments with sensor malfunctions or experimental errors (e.g., detached electrodes, incorrect electrode placement, wire disconnection), one participant was removed from each dataset (experimental and clinical), yielding 23 and 49 participants in the experimental and clinical datasets, respectively. Additionally, in the experimental dataset, one participant was excluded from the VM task, and a different participant was excluded from the TG task. The final analysis includes the segments summarized in Table 1. Numbers outside parentheses indicate segments, and numbers in parentheses indicate contributing subjects. Statistical analyses were performed at the segment level, with subject included as a random effect in linear mixed-effects models to account for repeated measurements (see Section 2.5).

### 2.3. Time-Series Data Processing

**Figure 2:**
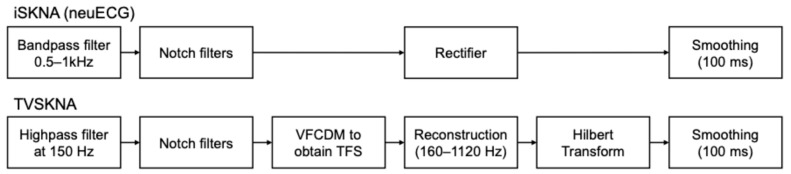
Data processing scheme for iSKNA and TVSKNA. VFCDM: variable frequency complex demodulation and TFS: time–frequency spectra.

In our analysis, two time-series SKNA measures were calculated using the pySKNA library 0.1.0 [30]: iSKNA and time-varying index of SKNA (TVSKNA). The implementation followed the algorithmic definitions described in this manuscript and prior neuECG publications [1,7,10]. Default parameter settings were used throughout, including the predefined filter ranges and smoothing window length, to ensure consistency with the established SKNA processing framework. Figure 2 describes flow charts illustrating the processing pipeline for both iSKNA and TVSKNA.

#### 2.3.1. Preprocessing

All signals were first downsampled from 10 kHz to 4 kHz using a polyphase resampling method [31], which applies an internal finite impulse response (FIR) anti-aliasing filter prior to decimation. The 4 kHz sampling rate was selected to preserve the known SKNA frequency range (150–1000 Hz) [10] while reducing computational burden.

The downsampled signals were subsequently filtered. Both iSKNA and TVSKNA were derived from the same downsampled ECG signals but processed through separate filtering pipelines consistent with their methodological definitions. For iSKNA, a 0.5–1 kHz bandpass FIR filter was designed using a Kaiser window (60 dB stopband attenuation), with filter order determined via the Kaiser design method. For TVSKNA, a 150 Hz highpass FIR filter (60 dB stopband attenuation) was applied prior to time–frequency decomposition. All FIR filters were applied using zero-phase forward–backward filtering to avoid phase distortion.

Narrowband interference frequencies were identified using power spectral density (PSD) analysis for each participant, with visual inspection used to confirm distinct, sharp spectral peaks. Frequencies that were consistently present in both baseline and task recordings within the same participant were considered condition-independent artifacts and removed using second-order IIR notch filters (Q = 30), applied with zero-phase filtering.

These preprocessing differences reflect the distinct conceptual representations of iSKNA and TVSKNA, and comparisons were performed within each index across conditions rather than between indices.

#### 2.3.2. Integrated SKNA

Using the neuECG technique, the iSKNA time-series data were obtained [7]. This technique has been widely used for SNS assessment due to its simplicity, intuitive implementation, and fast computation. The iSKNA time series are typically calculated as follows: (1) application of a bandpass filter with cutoff frequencies between 500 Hz and 1 kHz, (2) signal rectification, and (3) smoothing using a moving average filter with a 100 ms window. While various bandpass ranges have been used in previous studies, including 0.5–0.7 kHz, 0.2–1 kHz, 0.7–2 kHz, and 1.7–2 kHz [1,4,7,8,32], we used a 0.5–1 kHz range. This range is most commonly used in SKNA studies, and also showed high sensitivity in detecting VM and TG sensations [1,10].

#### 2.3.3. Time-Varying SKNA

We recently developed a new time-varying measure of SKNA designed to capture dynamic fluctuations of SKNA over time. This approach exhibited greater sensitivity and reliability in assessing SNS activity compared to iSKNA [10]. A study found that the majority of spectral power was concentrated in the low-frequency band (150–500 Hz), with a significant increase extending up to 1000 Hz. Based on these findings, we computed TVSKNA features by reconstructing the time–frequency spectra within the 160–1140 Hz range. The difference between the reported SKNA band (150–1000 Hz) and the reconstructed TVSKNA range (160–1120 Hz) results from the discrete frequency resolution of the VFCDM algorithm. As VFCDM generates components at fixed 160 Hz intervals, components from 160 to 1120 Hz were summed to approximate the target SKNA band.

We acknowledge concerns regarding muscle noise when analyzing frequency bands below 400 Hz. However, a recent study showed that muscle noise interference is not limited to frequencies below 400 Hz, but extends into the 0.5–1 kHz frequency band [33]. In that frequency band, 95% of the energy associated with muscle noise was found within the 508–898 Hz band.

**Figure 3:**
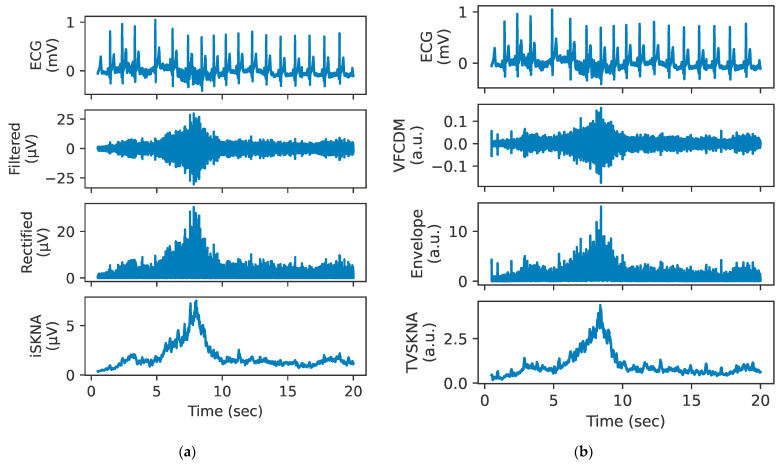
Example SKNA processing pipeline during the Valsalva maneuver (VM). The left panel (**a**) illustrates the iSKNA computation pipeline: bandpass filtering (500–1000 Hz), rectification, and temporal integration using a 100 ms moving-average filter (from top to bottom). The right panel (**b**) illustrates the TVSKNA computation pipeline: highpass filtering (150 Hz), VFCDM-based reconstruction of the high-frequency component, envelope extraction via the Hilbert transform, and temporal integration using a 100 ms moving-average filter (from top to bottom). VFCDM: variable frequency complex demodulation.

The computation of TVSKNA consists of three steps: (1) signal decomposition using variable frequency complex demodulation (VFCDM) [34] to obtain time–frequency spectrum (TFS) followed by reconstruction by summing of the TFS components, (2) estimation of the instantaneous amplitude using the Hilbert transform, and (3) smoothing using a moving average filter with a 100 ms window. VFCDM is an optimal technique for TFS analysis because it provides higher resolutions while retaining accurate amplitude estimates compared to other methods [34]. A brief summary of the method is provided in Appendix A, and additional methodological details are provided in our previous publication [10]. Figure 3 shows an example processing of both iSKNA and TVSKNA during VM.

### 2.4. Complexity Analysis

We conducted complexity analyses based on entropy indices, fractal dimensions, and Hjorth parameters.

#### 2.4.1. Approximate and Sample Entropy

Entropy is a measure of disorder, randomness, uncertainty, or complexity within a system. We computed approximate (ApEn) [35] and sample entropy (SampEn) [36] to quantify the complexity of physiological time-series data. These entropy measures have been widely applied in the analysis of EEG, fMRI, and HRV [37,38,39,40,41]. A brief summary of their computation is provided in Appendix B.

Both ApEn and SampEn have two important parameters, *m* and *r*, representing embedding dimension (or order) *m* and tolerance *r*, respectively. The embedding dimension defines the length of sequences to be compared, and the tolerance determines similarity between sequences. In our paper, *m* and *r* were set to 2 and 0.2 multiplied by the standard deviation of each signal, as these are most commonly recommended [42].

ApEn estimates the negative logarithm of the conditional probability that two similar sequences of length *m* remain similar when extended to length *m* + 1, with smaller values indicating more regular signals. SampEn differs by excluding self-matching and is therefore always non-negative.

#### 2.4.2. Hjorth Parameters

Hjorth parameters, including activity, mobility, and complexity, were originally proposed for analyzing EEG signals [43]. Since then, these parameters have been used to assess neurophysiological conditions in EEG signals, including Alzheimer’s disease [44], seizure lateralization [45] and other states [46,47,48]. Hjorth parameters are computed as follows:

(1) $$Activity=var(x(t))=\;\frac{\sum {\left(x_{i}-\mu \right)}^{2}}{N},$$

(2) $$Mobility=\sqrt{\frac{var(\frac{dx(t)}{dt})}{var(x\left(t\right))}},$$

(3) $$Complexity=\sqrt{\frac{Mobility(\frac{dx(t)}{dt})}{Mobility(x\left(t\right))}},$$

where *x*(*t*) represents the SKNA signals. Since our indices were compared with standard deviation (see Section 2.4.4), we only analyzed mobility and complexity to avoid redundancy. Hjorth activity was not analyzed separately because it is mathematically equivalent to signal variance. Since standard deviation (vSKNA) was already included as a benchmark linear measure (see Section 2.4.4), inclusion of Hjorth activity would have been redundant. Therefore, only Hjorth mobility and complexity were evaluated.

#### 2.4.3. Katz Fractal Dimension (KFD)

Fractal analysis is often used in neuroscience to solve complex patterns in neurophysiological signals, such as EEG and MRI [49]. In our work, we selected the KFD method as a representative index of fractal analysis, because it is known to be less sensitive to noise compared to other methods [49,50]. KFD is computed as follows:

(4) $$KFD\;=\frac{\log_{10}\left(n\right)}{\log_{10}\left(\frac{d}{L}\right)+\log_{10}\left(n\right)},$$

where *n*, *d*, and *L* represent the number of points, the maximum distance between the first point and any other point, and the sum of the Euclidean distances between successive points. The lower KFD values represent smoother and more linear signals.

In our paper, KFD was computed directly from the preprocessed SKNA segments without additional smoothing or amplitude normalization beyond the filtering steps described in Section 2.3.1. Identical preprocessing was applied across all conditions.

#### 2.4.4. Compared Index: Standard Deviation

As a benchmark linear measure, we calculated the standard deviation (S.D.) of each SKNA segment, as it has demonstrated high sensitivity and strong discriminative ability in various SNS conditions [1,10,11]. The inclusion of S.D. allowed comparison between conventional amplitude-based metrics and nonlinear complexity indices. Standard deviation was computed independently for each segment without cross-subject normalization, as between-subject variability was accounted for using mixed-effects modeling (see Section 2.5). Note that the standard deviation of iSKNA is commonly referred to as vSKNA; however, in this study, we report it as iSKNA S.D. for consistency with other indices.

### 2.5. Statistics

The VM segment size was set to 20 s (corresponding to 80 k samples per segment) to capture the inhale and exhale dynamics across all participants. For TG and cold test conditions, a shorter segment length of 5 s (corresponding to 20 k samples per segment) was used, as the majority of pain duration occurred within 5 s. Given the high sampling rate, both segment lengths provided sufficient data points for stable estimation of entropy and complexity measures. In addition, segment lengths longer than 5 s may include parasympathetic recovery effects following the initial sympathetic response. All comparisons were performed within each task using consistent segment durations across conditions.

For the significance test, we fitted linear mixed-effects models with a random intercept for participants using the LMER function in R (lmer package) [51], as each participant had multiple measurements, as follows:

(5) $$SKNA\;indices\;\sim \;Condition+\left(1\;\right|Participant),$$

where Participant was included as a random intercept.

Estimated marginal means (i.e., least square means) from the fitted LMER models were then obtained to perform a pairwise post hoc test, using R’s emmeans function with the Tukey method [52] if the one-way analysis of variance (ANOVA) via the LMER function was significant. No additional global correction was applied across indices, as the indices were analyzed as separate outcomes, and results were interpreted based on consistency across measures rather than isolated *p*-values. From LMER models, Cohen’s *d* was also calculated to quantify effect size and interpreted according to Sawilowsky’s criteria, with corresponding 95% confidence intervals [53]. We also investigated the effects of sex (factor) and age (covariate) using LMER, as follows:

(6) $$SKNA\;indices\;\sim \;Condition\;\times \;Sex+Age+\left(1\;\right|Participant),$$

where Participant was included as a random intercept.

Additionally, parameter sensitivity analyses were performed for ApEn and SampEn to evaluate whether discrimination performance was influenced by condition, embedding dimension (*m*), tolerance (*r*), or their interactions. Linear mixed-effects models were specified as

(7) $$Entropy\;indices\;\sim \;Condition\times m\times r+\left(1\;\right|Participant),$$

where Participant was included as a random intercept. To evaluate pairwise differences in discrimination across parameter combinations, estimated marginal means were obtained using the emmeans package. Because multiple contrasts were tested across parameter settings, false discovery rate (FDR) correction was applied to adjust for multiple comparisons. Effect sizes were quantified using Cohen’s *d* with 95% confidence intervals.

Model assumptions were evaluated for all mixed-effects models through visual inspection of residual diagnostics. Normality was assessed using Q–Q plots, and homoscedasticity was examined using residual-versus-fitted plots. No substantial violations were observed. We also evaluated models with random slopes for condition; however, these models frequently resulted in singular fits, likely due to limited repeated observations per participant and an unbalanced data structure. Therefore, random-intercept models were retained as the most parsimonious and statistically stable specification.

Additionally, ROC analyses were conducted to quantify the discriminative performance of individual indices and did not involve predictive model training or cross-validation procedures. ROC curves were generated separately for each SKNA index to evaluate their ability to distinguish between predefined condition groups [54]. ROC curves calculate true positive and false positive rates of each index at all possible decision thresholds that determine two classes (Class 1 and Class 0). In this paper, class 1 was set to SNS tasks with higher index values for fair comparisons. To evaluate the ROC curves, AUCs were computed. AUC values range between 0 and 1. The higher AUC indicates a more discriminative ability to detect SNS activity. An AUC of 0.5 is considered no discrimination. Values between 0.7 and 0.8 are considered acceptable, 0.8 to 0.9 are considered excellent, and values ≥ 0.9 are regarded as outstanding [54]. Confidence intervals for AUC were computed using DeLong’s method [55]. Using the pROC package [56], DeLong’s test was used to compare AUC values and determine whether two correlated ROC curves had significantly different AUCs [55].

Finally, we computed repeated-measures correlation coefficients and their corresponding 95% confidence intervals between VAS and each SKNA index using the RMCORR package in R [57], which accounts for within-subject dependence. Interpretation of the correlation coefficients followed established guidelines, in which values of 0.3–0.5 are considered fair, 0.6–0.7 moderate, and 0.8–0.9 very strong [58].

We considered a *p*-value < 0.05 to be statistically significant.

## 3. Results

**Figure 4:**
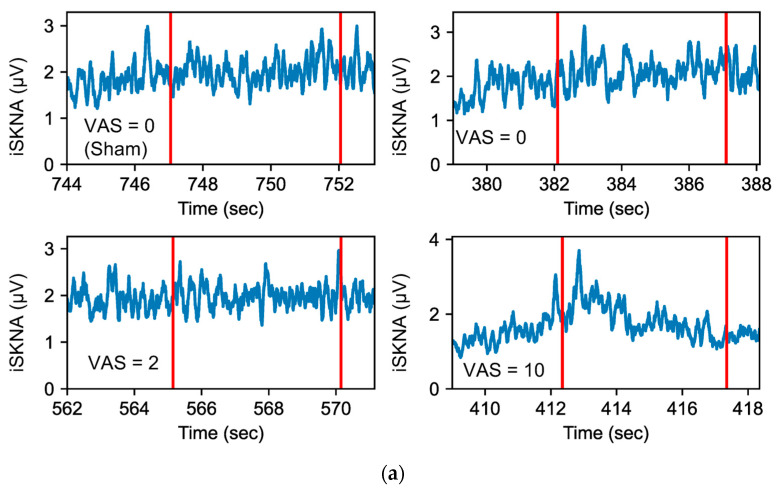

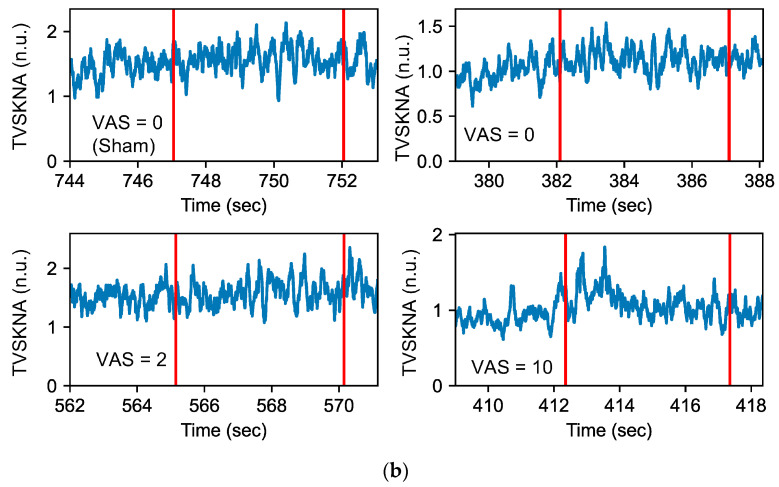
Examples of iSKNA (**a**) and TVSKNA (**b**) signals of one subject (3 teeth) with different VAS scores during cold test; red lines indicate the start and end points of cold test.

Figure 4 illustrates representative iSKNA and TVSKNA signals from a single participant during the cold test under varying pain intensities. Visual inspection shows increased signal variability during higher VAS scores (e.g., VAS = 10) compared to no pain or low-pain conditions. These qualitative changes motivated the quantitative analyses presented below.

### 3.1. Experimental Dataset

We found that complexity analysis is a statistically effective approach for detecting short-term SNS activity using SKNA in healthy participants. Specifically, both TVSKNA and iSKNA indices showed significant differences for VM (*p* < 0.001; Table S1). For iSKNA, effect sizes were large, with Cohen’s *d* = 3.03 (95% CI: 2.42–3.64) for standard deviation and 2.80–3.46 for complexity measures (highest for SampEn). For TVSKNA, effect sizes were slightly smaller but remained large (Cohen’s *d* = 1.97 [95% CI: 1.46–2.47] for standard deviation; 1.93–2.65 for complexity measures, highest for ApEn). All complexity indices demonstrated excellent-to-outstanding AUC values (0.86–0.96; Table S2).

TG tasks exhibited significant differences compared with NP (*p* < 0.001) and between TG CSP− vs. CSP+ groups (*p* < 0.05) (Table S2 and Figures S1 and S2). Both TVSKNA and iSKNA indices demonstrated large-to-huge effect sizes for NP vs. CSP+ (|*d*| = 1.46–2.11, Table 2). Standard deviation showed similarly large effects within this comparison (Cohen’s *d* = 2.05 [95% CI: 1.5–2.59] for iSKNA and 2.22 [95% CI: 1.67–2.77] for TVSKNA; see Table S1). Effect sizes were smaller for CSP− vs. CSP+, reflecting reduced sensitivity for distinguishing adjacent pain levels. In this comparison, entropy measures showed moderately large effects (|*d*| = 0.63–0.71; see Table S1), whereas S.D. and mobility were moderate (|*d*| = 0.58–0.68; see Table S1), and Hjorth Complexity was small-to-moderate (|*d*| = 0.40–0.45; see Table S1).

This graded pattern was consistent with the ROC analysis. Both signals achieved high AUC values for NP vs. CSP− and NP vs. CSP+, with complexity features ranging from 0.80 to 0.92 and S.D. from 0.95 to 0.99. In contrast, discrimination between CSP− and CSP+ was limited (complexity: 0.56–0.64; S.D.: 0.63).

Interestingly, mobility, KFD, and entropy indices were higher during the baseline compared to each SNS task, and also higher during CSP− compared to CSP+. This pattern was also observed in repeated-measures correlations analyses with VAS, which showed fair-to-moderate associations (complexity: |*r*| = 0.42–0.56; S.D.: 0.56–0.60; *p* < 0.001), with negative associations observed for all complexity-based indices (except for Hjorth Complexity) and positive associations for S.D. (Table S3). The direction of correlation reflects both the mathematical definition and physiological behavior of each metric. Increased pain was associated with higher signal amplitude (e.g., increased S.D.) but reduced irregularity (e.g., decreased entropy and related measures), consistent with more structured sympathetic activity. Interestingly, Hjorth Complexity, which reflects relative changes in frequency content rather than signal irregularity alone, showed a different directional pattern. Thus, negative correlations for nonlinear indices do not indicate reduced SNS activation, but rather increased signal regularity at higher pain levels. This interpretation is further discussed in the Section 4.

### 3.2. Clinical Dataset

In the clinical dataset, discrimination was strongest for CSP+ vs. NP, with large effect sizes observed across both iSKNA and TVSKNA signals (complexity features: |*d*| = 0.86–1.12; S.D.: |*d*| = 1.01–1.17; Table 3). Separation between CSP− and CSP+ was moderate (complexity: |*d*| = 0.63–0.77; S.D.: |*d*| = 0.87–0.90), whereas NP vs. CSP− showed small-to-moderate effects (complexity: |*d*| = 0.24–0.46; S.D.: |*d*| = 0.14–0.27), indicating reduced sensitivity for distinguishing lower pain levels. Compared with the experimental dataset, overall effect sizes were smaller, consistent with greater heterogeneity in the clinical population.

ROC analyses reflected this graded pattern. For NP vs. CSP+, discrimination ranged from acceptable to excellent in the severe-anxiety (SA) group (complexity: AUC 0.70–0.96; S.D.: 0.82–0.86), whereas performance was lower in the non-severe-anxiety (NSA) group (complexity: 0.64–0.69; S.D.: 0.74–0.75). Similarly, for CSP− vs. CSP+, discrimination was limited in the NSA group (complexity: 0.49–0.56; S.D.: 0.71–0.74) but higher in the SA group (complexity: 0.70–0.91; S.D.: 0.71–0.83).

Discrimination between NP and CSP− remained limited in both SA and NSA groups (complexity: AUC 0.42–0.66; S.D.: 0.49–0.62). Notably, several indices demonstrated significantly higher AUC values in the SA group compared with the NSA group (*p* < 0.05; Table S5), suggesting enhanced separability in individuals with higher anxiety levels. However, because the SA group included fewer participants (Table 1), this exploratory analysis should be interpreted with caution.

Finally, repeated-measures correlation analyses showed fair-to-moderate associations between VAS and SKNA indices. In the SA group, correlations ranged from |*r*| = 0.51–0.72 for complexity features and 0.49–0.50 for S.D., whereas in the NSA group, they ranged from |*r*| = 0.38–0.50 for complexity features and 0.66–0.70 for S.D. As observed in the experimental dataset, most complexity measures exhibited negative associations with VAS, while Hjorth’s complexity and S.D. showed positive associations (Table S6).

### 3.3. Entropy Parameter Sensitivity

**Figure 5:**
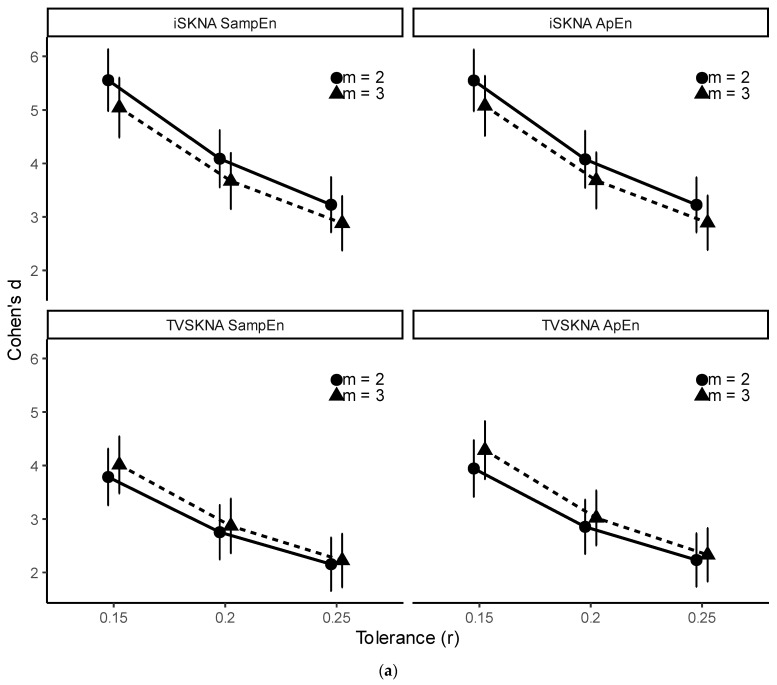

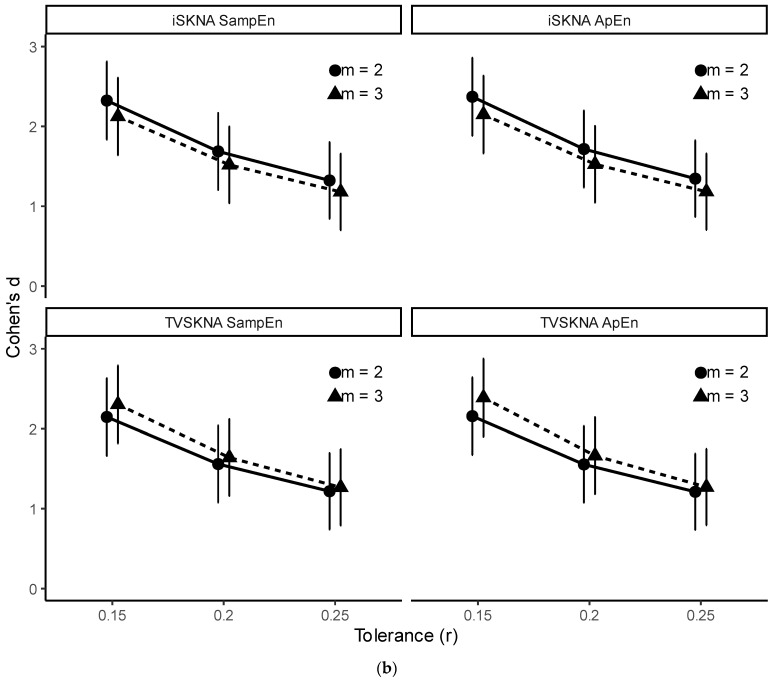

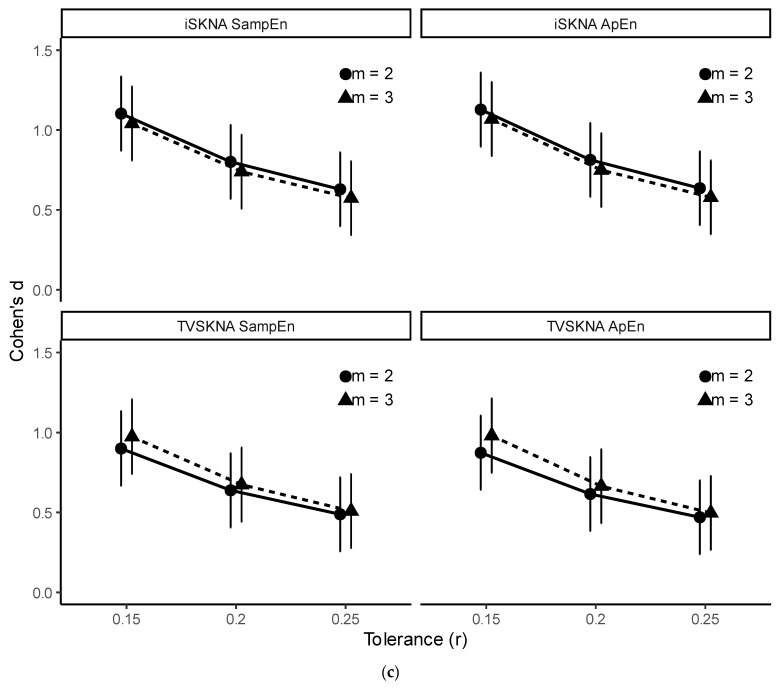
Parameter sensitivity of entropy-based discrimination, expressed as Cohen’s *d* between (**a**) baseline vs. Valsalva maneuver (VM); (**b**) NP vs. CSP+ (experimental dataset); and (**c**) NP vs. CSP+ (clinical dataset). Error bars indicate 95% confidence intervals.

We evaluated the sensitivity of ApEn and SampEn to embedding dimension (*m* = 2, 3) and tolerance (*r* = 0.15, 0.20, 0.25 × SD). The impact of embedding dimension (*m*) was minimal overall, with the exception of iSKNA-derived entropy during VM (described below). In contrast, discrimination strength differed significantly across tolerance values, with the largest effect sizes observed at lower *r* (Figure 5).

In the experimental–VM dataset, both ApEn and SampEn exhibited significant Condition × *r* interactions for iSKNA (*p* < 0.001) and TVSKNA (*p* < 0.001), indicating that discrimination strength varied across tolerance values. A significant Condition × *m* interaction was observed only for iSKNA (*p* < 0.05). Post hoc comparisons demonstrated robust baseline vs. VM differences across all tolerance levels and embedding dimensions for both iSKNA- and TVSKNA-derived entropy measures (all *p* < 0.001; Figures S5 and S6).

In the experimental–TG dataset, both ApEn and SampEn exhibited significant Condition × *r* interactions for iSKNA and TVSKNA (ApEn: *p* < 0.001; SampEn: *p* < 0.05). Post hoc comparisons revealed significant NP vs. non-NP differences across all parameter combinations (*p* < 0.001), except for iSKNA ApEn at *m* = 3 and *r* = 0.25 for the NP vs. CSP− contrast (*p* < 0.05) (Figures S7 and S8).

In the clinical dataset, both ApEn and SampEn demonstrated a significant Condition × *r* interaction for iSKNA (*p* < 0.001) and TVSKNA (*p* < 0.05), indicating that discrimination strength varied across tolerance values. Post hoc comparisons revealed CSP+ vs. non-CSP+ differences across all tolerance levels for iSKNA-derived entropy measures (*p* < 0.001; see Figure S9). For TVSKNA, significant pairwise differences were observed across all tolerance levels for *r* ≤ 0.20 (*p* < 0.05). At *r* = 0.25, significant discrimination was primarily observed for CSP+ vs. non-CSP+ groups, except for ApEn at *m* = *2*, where NP vs. CSP+ did not reach statistical significance (Figures S9 and S10).

### 3.4. Effects of Sex and Age

Our LMER analyses also showed no statistically significant effects of either sex or age in both datasets. Note that age did not differ significantly between males and females (Table 4).

## 4. Discussion

This study demonstrates that magnitude-based (S.D.) and complexity-based SKNA metrics provide complementary characterization of sympathetic activation across experimental and clinical contexts. S.D. was particularly sensitive to large amplitude shifts, such as those observed during VM and NP vs. CSP+ contrasts, reflecting changes in overall signal dispersion. In contrast, entropy- and fractal-based measures captured graded alterations in signal irregularity, particularly in adjacent pain comparisons and in exploratory anxiety-stratified ROC analyses. Importantly, neither feature class uniformly dominated; rather, their relative performance depended on the physiological contrast examined. Together, these findings suggest that combining magnitude- and complexity-based descriptors offers a more comprehensive representation of SKNA dynamics during SNS activation.

### 4.1. Physiological Interpretation of Reduced SKNA Complexity During Sympathetic Activation

One interesting finding is that most complexity features showed higher values at lower levels of stimulation (i.e., the baseline vs. stimulation or CSP− vs. CSP+), except for Hjorth’s complexity. Consistent with this pattern, our repeated-measures correlation analyses showed negative correlations for these indices (Tables S3 and S6). Other SKNA studies, including our previous works, have presented increased SKNA variability and amplitude during SNS activation, consistent with the standard deviation analysis reported in this paper [1,2,3,4,7,8,10,11,32].

Similar entropy and complexity reductions under stress or task engagement have been observed in other physiological modalities. For example, Barzegar et al. reported decreased KFD, SampEn, and ApEn in EEG signals during social stress, along with increased delta-band power [59]. Decreased ApEn has also been observed in delta-band EEG signals during an arithmetic task load [60]. In fMRI studies, SampEn of blood oxygenation level-dependent signals decreased during a cognitive-memory task compared to the resting state, which was interpreted as reflecting increased neural coordination [61]. Likewise, reduced entropy features have been reported in HRV during stress-inducing tasks [62,63,64], demonstrating directional reductions in physiological complexity under sympathetic dominance. Although direct numerical comparison across modalities is limited by differences in signal properties and preprocessing methods, the magnitude and direction of complexity reductions observed in SKNA during VM and high-pain contrasts (|*d*| up to 2–3 in the experimental dataset) are consistent with reports of reduced physiological complexity during acute sympathetic activation.

While EEG, fMRI, HRV, and SKNA measure distinct physiological processes with different spatial and temporal characteristics, converging evidence suggests that stress and task-related SNS activation are often accompanied by reduced signal complexity, potentially reflecting increased coordination or more constrained temporal organization of physiological activity. Nevertheless, modality-specific factors may influence effect magnitude and discriminability, and such patterns should not be assumed to generalize universally without direct validation.

Taken together, our findings suggest that SKNA may exhibit more coordinated or constrained temporal organization during SNS activation compared to resting states, consistent with—but not interchangeable with—observations in other physiological systems.

### 4.2. Contextual-Dependent and Anxiety-Related Differences in SKNA Responses

Our analyses indicate that the experimental and clinical datasets exhibited distinct structural complexity patterns. Effect size and AUC analyses revealed that the TG condition did not show clear discriminability between CSP− and CSP+, whereas in the clinical dataset, NP and CSP− were similarly indistinguishable. One possible explanation is contextual stress. In the clinical dataset, NP segments were recorded during a dental procedure setting, where anticipatory anxiety or procedural context may elevate SNS activity even in the absence of pain. In contrast, TG baseline segments were recorded under controlled resting conditions prior to stimulation, with participants informed that no tissue injury would occur and able to withdraw at any time.

Importantly, within the clinical dataset, SKNA AUC values were markedly higher in the SA group compared to the NSA group, suggesting that anxiety may modulate pain-related sympathetic responses. Severe anxiety is associated with heightened activation of the central autonomic network, including the amygdala, anterior cingulate cortex, insula, and hypothalamus [65,66]. These regions facilitate increased sympathetic outflow via hypothalamic and brainstem autonomic centers, leading to elevated baseline sympathetic tone and exaggerated reactivity to noxious stimuli [67,68].

Because SKNA reflects postganglionic sympathetic nerve discharges innervating sweat glands and cutaneous vasculature [8,69], heightened central arousal in SA individuals may lower the threshold for sympathetic activation and prolong sympathetic bursts. Consequently, pain-related stimuli may evoke stronger and more sustained sympathetic firing, resulting in larger SKNA amplitudes and higher AUC values. Moreover, anxiety and pain share overlapping limbic and salience-processing networks [70,71]. Heightened anticipatory threat processing in SA participants may increase perceived nociceptive salience, thereby augmenting autonomic responses even when objective pain intensity is similar. Together, these mechanisms likely contribute to the enhanced SKNA AUC observed in the SA group.

### 4.3. Selection of Reference and Complexity Measures

To contextualize the complexity analyses, we selected S.D. as a representative magnitude-based index, reflecting the burst-like morphology and amplitude variability of SKNA. Other SKNA indices have been proposed to quantify autonomic function, including the SKNA energy ratio (SKNAER) between bursts and non-bursts of SKNA signals [3,32] and SKNA drivers [2], which has shown high discriminatory powers compared to mean and S.D. of iSKNA. However, these approaches require clear bursts of SKNA for accurate detection of bursts, which were not reliably present in our clinical datasets (see Figure 4).

Cold testing elicits different pulpal responses: no sensation in necrotic teeth, mild sensation in normal teeth, and severe pain in pulpitis. The latter is more strongly associated with acute SNS activation compared to the others. This may explain the large effect sizes between CSP+ and the others in our clinical dataset, whereas effect sizes between NP and CSP− were small to medium. Note that SKNAER and the SKNA driver were developed using datasets involving relatively strong SNS activation, such as cold pressor testing (associated with intense pain), VM (strong baroreflex stress), and hemodialysis. These tasks and conditions are known to elevate muscle sympathetic nerve activity [18,72,73,74]. These indices may not be optimal for applications involving mild sensory stimuli, such as those in our clinical dataset.

Regarding entropy-based analyses, several alternative measures exist, such as permutation entropy, spectral entropy, and singular value decomposition (SVD) entropy. In this study, ApEn and SampEn were selected a priori because they are among the most widely used entropy indices for short neurophysiological time-series signals and have well-established parameter conventions. Their foundation in time-series analysis makes them particularly well-suited for quantifying the temporal irregularity and structural organization of SKNA within each analysis window. Prior work has also demonstrated strong performance of ApEn and SampEn in detecting burst suppression compared to other entropy measures [75]. However, the performance of entropy indices can vary depending on the application [76].

In addition to the primary complexity measures reported in the main analysis, we evaluated other entropy- and fractal-based measures, including permutation entropy [77], spectral entropy [78], SVD entropy [79], detrended fluctuation analysis [80], Higuchi fractal dimension [81], and Petrosian fractal dimension [82] (Table S7). While several alternative measures demonstrated significant effects in isolated comparisons, their effect sizes were less consistent across signal types (iSKNA vs. TVSKNA) and experimental paradigms, and confidence intervals frequently crossed zero. In contrast, the primary complexity indices showed more stable effect size magnitudes and consistent directional patterns across datasets.

### 4.4. Relation to Prior Work

Our experimental dataset was collected from twenty-four participants, of whom sixteen were included in our previous publications [1,2]. In our earlier analysis [83], we focused only on iSKNA using the descriptive indices, including maximum, mean, and standard deviation. VM exhibited a similar AUC value; however, the TG data in that study used a different baseline definition. In the present study, our analyses were based on VAS, as it is the clinical standard and allows us to compare performance across the two datasets. In our previous work, we analyzed classification performance based on stimulation levels, which generally yielded higher AUC values (AUC of 0.90–1). This phenomenon—where classification performance is higher when using stimulation intensity rather than VAS—has also been observed in electrodermal-activity-based studies [84].

### 4.5. Entropy Parameters

Both ApEn and SampEn rely on two key parameters: the embedding order (*m*) and the tolerance (*r*). In this study, we used an embedding order of 2 and a tolerance set to 0.2 times the standard deviation of each signal. This configuration is commonly used and is the default in many software libraries [85,86,87]. Researchers have emphasized the importance of these parameters, as suboptimal choices may fail to distinguish between noise and meaningful signal patterns [88].

Importantly, parameter sensitivity analyses revealed statistically significant effects of tolerance (*r*) and, to a lesser extent, embedding dimension (*m*) on discrimination performance (Figure 5 and Figures S5–S10). Lower tolerance values were associated with larger effect sizes, indicating that discrimination strength varied systematically across parameter settings. Nevertheless, most condition contrasts remained significant across most parameter combinations.

From a translational perspective, these findings suggest that although entropy magnitude and effect size depend on parameter selection, the overall discriminative capacity of SKNA-derived entropy measures is preserved within a practical parameter range. In real-time or wearable applications, such consistency may enhance reproducibility while still allowing application-specific parameter optimization.

### 4.6. Computation Time

Regarding computation time, ApEn and SampEn have a time complexity of O(*N*^2^), which can be improved to O(NlogN) using the KD-tree algorithm; however, in the worst-case scenario, the complexity may still reach O(*N*^2^). In contrast, other complexity analysis indices have a complexity of O(*N*). Given the high sampling frequency required for SKNA signals, the use of entropy-based indices may significantly prolong the execution time of the analysis. Fast computation algorithms for estimating SampEn and ApEn [89,90] may help address this issue and warrant further exploration in future studies, particularly in applications involving wearable devices where real-time computation is desired. In contrast, the Hjorth mobility index may be more suitable for real-time SKNA analysis, as it offers comparable performance to entropy indices with lower computational cost.

### 4.7. Limitations

Despite the promising results of our complexity analysis, there are several limitations to this study.

#### 4.7.1. High Sampling Frequency and Noise Susceptibility

A major concern is that SKNA requires a high sampling frequency—in our case, 4 kHz—which increases susceptibility to unwanted noise, such as interference from nearby electronic devices. We manually identified and filtered out these noise components, but only when they were present in both the baseline and SNS task segments. Future studies should aim to develop automated algorithms for detecting and removing such noise frequencies to ensure more accurate assessments.

#### 4.7.2. Motion and EMG Contamination

Another important limitation involves potential motion and muscle artifacts. The frequency range of SKNA (150–1000 Hz) overlaps partially with surface EMG activity. Although early neuECG methodology assumed predominant muscle activity below 400 Hz, more recent evidence [33] indicates that EMG contamination may extend into the 0.5–1 kHz band. In our previous work, we observed that SKNA spectral power was primarily concentrated between 150 and 500 Hz [10]. While EMG power generally decreases at higher frequencies, low-amplitude residual EMG components or spectral leakage may still appear in this range and contaminate SKNA features.

Such contamination could influence the effect size and AUC estimates. Random motion may increase within-group variability and attenuate discrimination, whereas condition-related muscle tension could inflate SKNA amplitude and exaggerate between-group differences. In this study, participants were seated and instructed to minimize movement, visually evident artifacts were excluded, and spectral filtering was applied. Nevertheless, subtle EMG contamination cannot be fully excluded. Future studies incorporating simultaneous EMG monitoring or automated artifact-detection algorithms may further enhance specificity.

#### 4.7.3. Segment-Level ROC Analysis

ROC/AUC values were computed at the segment level to quantify discriminative performance. Because segments from the same participant are not fully independent, confidence intervals may be slightly optimistic; however, inferential comparisons were conducted using mixed-effects models that accounted for within-subject dependence.

#### 4.7.4. Electrode Configuration Variability

ECG lead configuration differed across participants and datasets (wrist–wrist vs. wrist–contralateral ankle), reflecting practical constraints in the experimental and clinical settings. Because SKNA extraction may be influenced by electrode placement, variability in lead configuration could affect amplitude and noise characteristics. Although analyses were performed within datasets and did not rely on direct amplitude comparisons across configurations, future studies should standardize electrode placement to minimize potential variability.

#### 4.7.5. VM Strain Intensity

Because intrathoracic pressure was not directly monitored during VM, variability in strain intensity across participants may have influenced the magnitude of sympathetic activation.

#### 4.7.6. Smoothing Window

Both iSKNA and TVSKNA include a 100 ms moving average as part of their established computational definitions. This smoothing step was applied identically across all conditions and participants. Because comparisons were performed within each index across experimental conditions rather than between differently processed signals, any influence of the smoothing window would affect all conditions equally and is unlikely to account for the observed condition-dependent differences. The 100 ms window length was adopted from prior neuECG methodology [7] and reflects a balance between temporal resolution and noise reduction. Future work may explore sensitivity to alternative smoothing windows; however, the present implementation follows established SKNA processing protocols.

## 5. Conclusions

In conclusion, magnitude-based and complexity-based SKNA metrics provide complementary characterization of sympathetic activation in both experimental and clinical settings. While S.D. reflects amplitude dispersion, entropy- and mobility-based measures capture structured changes in temporal organization, with TVSKNA approximate entropy and Hjorth’s mobility showing consistent sensitivity across datasets. These findings suggest potential utility for objective assessment of short-term sympathetic arousal in stress- and pain-related contexts. However, interpretation should consider the modest sample size, high sampling frequency requirements, and susceptibility to motion and EMG contamination. Future studies should validate these findings in larger and more diverse populations, compare SKNA complexity measures with established autonomic indices, and evaluate feasibility in real-time or wearable implementations.

## Figures and Tables

**Table 1:** Number of segments for datasets. The numbers in the parentheses indicate the number of subjects.

	**Baseline**	**SNS Stimulation**	**Segment Size**
VM	22 (22)	90 (22)	20 s
	**Baseline**	**CSP− and CSP+**	**Segment Size**
TG	22(22)	22 (11) and 110 (22)	5 s
Cold test	91 (48)	57 (29) and 86 (38)	5 s
Cold test (DAS ≥ 15)	10 (5)	13 (6) and 10 (5)	5 s

DAS: Corah’s Dental Anxiety Scale (scores ≥ 15 indicate severe anxiety).

**Table 2:** Summary of discriminative performance (TG—experimental dataset).

		|*d*|		AUC	
		S.D.	Complexity (Range; Highest Metric)	S.D.	Complexity (Range; Highest Metric)
iSKNA	NP vs. CSP−	1.47	1.06–1.53 (KFD)	0.95	0.81–0.92 (KFD)
	NP vs. CSP+	2.05	1.46–2.10 (KFD)	0.95	0.83–0.90 (KFD)
	CSP− vs. CSP+	0.58	0.4–0.64 (ApEn)	0.63	0.56–0.62 (Mobility)
TVSKNA	NP vs. CSP−	1.64	1.01–1.42 (ApEn)	0.99	0.80–0.83 (ApEn)
	NP vs. CSP+	2.22	1.47–2.11 (ApEn)	0.99	0.84–0.86 (Complexity)
	CSP− vs. CSP+	0.58	0.45–0.71 (SampEn)	0.63	0.61–0.64 (KFD)

**Table 3:** Summary of discriminative performance (clinical dataset).

		|*d*|		AUC (NSA)		AUC (SA)	
		S.D.	Complexity	S.D.	Complexity	S.D.	Complexity
			(Range; Highest Metric)		(Range; Highest Metric)		(Range; Highest Metric)
iSKNA	NP vs. CSP−	0.14	0.24–0.37 (Mobility)	0.49	0.64–0.66 (Complexity)	0.62	0.46–0.51 (KFD)
	NP vs. CSP+	1.01	0.86–1.12 (Mobility)	0.74	0.64–0.69 (ApEn)	0.82	0.81–0.87 (Mobility)
	CSP− vs. CSP+	0.87	0.63–0.77 (Complexity)	0.74	0.53–0.56 (Mobility)	0.71	0.81–0.82 (Complexity)
TVSKNA	NP vs. CSP−	0.27	0.25–0.46 (Mobility)	0.53	0.60–0.66 (Mobility)	0.55	0.42–0.53 (Complexity)
	NP vs. CSP+	1.17	0.72–1.04 (Mobility)	0.75	0.59–0.67 (ApEn)	0.86	0.7–0.96 (SampEn)
	CSP− vs. CSP+	0.9	0.47–0.58 (Complexity)	0.71	0.49–0.51 (ApEn)	0.83	0.7–0.91 (SampEn)

**Table 4:** Age distribution between males and females.

	Male	Female	*p*-Value (*t*-Test)
Experimental Data	25.0 ± 3.9	30.1 ± 10.6	*p* = 0.16 (n.s.)
Clinical Data	38.5 ± 15.1	39.8 ± 13.0	*p* = 0.94 (n.s.)

## Data Availability

The data that support the findings of this study are available from the corresponding author upon reasonable request.

## Supplementary Materials

The following supporting information can be downloaded at: https://www.mdpi.com/article/10.3390/s26051611/s1, Table S1: Effect size (Cohen’s *d*) for the experimental dataset (95% CI); Table S2: AUC for the experimental dataset (95% CI); Table S3: Repeated measures correlation coefficients for experimental dataset (95% CI); Table S4: Effect size (Cohen’s *d*) for clinical dataset (95% CI); Table S5: AUC values for non-severe-anxiety (NSA) and severe-anxiety (SA) groups from clinical dataset (95% CI); Table S6: Repeated measures correlation coefficients for clinical dataset (95% CI); Table S7: Cohen’s *d* (95% CI) for additional entropy and complexity measures; Figure S1: Boxplots for (a) iSKNA and (b) TVSKNA during VM; Figure S2: Boxplots for (a) iSKNA and (b) TVSKNA during thermal grill (TG) pain; Figure S3: Boxplots for (a) iSKNA and (b) TVSKNA during cold test; Figure S4: Boxplots for iSKNA (a) and TVSKNA (b) between non-severe and severe anxiety groups from clinical dataset. None of indices exhibited significant difference between those two groups. However, the severe anxiety group showed greater discriminatory between CSP+ and the other levels (See Table S5); Figure S5: Parameter sensitivity of entropy-based discrimination from iSKNA during the Valsalva maneuver. (a) Sample entropy and (b) Approximate entropy. * *p* < 0.05, ** *p* < 0.001 (FDR-adjusted); Figure S6: Parameter sensitivity of entropy-based discrimination from TVSKNA during the Valsalva maneuver. (a) Sample entropy and (b) Approximate entropy. * *p* < 0.05, ** *p* < 0.001 (FDR-adjusted); Figure S7: Parameter sensitivity of entropy-based discrimination from iSKNA during the thermal grill stimulation. (a) Sample entropy and (b) Approximate entropy. * *p* < 0.05, ** *p* < 0.001 (FDR-adjusted); Figure S8: Parameter sensitivity of entropy-based discrimination from TVSKNA during the thermal grill stimulation. (a) Sample entropy and (b) Approximate entropy. * *p* < 0.05, ** *p* < 0.001 (FDR-adjusted); Figure S9: Parameter sensitivity of entropy-based discrimination from iSKNA during cold test. (a) Sample entropy and (b) Approximate entropy. * *p* < 0.05, ** *p* < 0.001 (FDR-adjusted); Figure S10: Parameter sensitivity of entropy-based discrimination from TVSKNA during cold test. (a) Sample entropy and (b) Approximate entropy. * *p* < 0.05, ** *p* < 0.001 (FDR-adjusted).

## Abbreviations

The following abbreviations are used in this manuscript:

ECG	Electrocardiogram
SKNA	Skin nerve activity
VM	Valsalva Maneuver
TG	Thermal Grill
VAS	Visual analog scale
AUC	Area under the curve
SNS	Sympathetic nervous system
SSNA	Skin sympathetic nerve activity
iSKNA	Integrated SKNA
TVSKNA	Time-varying SKNA
DAS	Corah’s Dental Anxiety Scale
ApEn	Approximate entropy
SampEn	Sample entropy
KFD	Katz fractal dimension
EEG	Electroencephalogram
MRI	Magnetic Resonance Imaging
ROC	Receiver operating characteristic
NP	No pain
CSP−	Clinically non-significant pain
CSP+	Clinically significant pain
NSA	Non-severe anxiety
SA	Severe anxiety
VFCDM	Variable frequency complex demodulation
TFS	Time–frequency spectra
HIPAA	Health Insurance Portability and Accountability Act
FIR	Finite impulse response
PSD	Power spectral density
LPF	Low-pass filter
FDR	False discovery rate
ANOVA	Analysis of variance

## Appendix A

First, the ECG signal *x*(*t*) is considered as a narrow band oscillation with a center frequency *f_0_*, instantaneous amplitude *A*(*t*), phase *φ*(*t*), and the direct current component *dc*(*t*), as follows:

(A1) $$x\left(t\right)=dc\left(t\right)+A\left(t\right)cos(2\pi f_{0}t+\varphi (t))$$

For a given center frequency, the instantaneous amplitude information *A*(*t*) and phase information *φ*(*t*) were extracted by multiplying Equation (A1) by $e^{-j2\pi f_{0}t}$, as follows:

(A2) $$z\left(t\right)=dc\left(t\right)e^{-j2\pi f_{0}t}+\frac{A(t)}{2}e^{j\varphi (t)}+\frac{A(t)}{2}e^{-j(4\pi f_{0}t+\varphi \left(t\right))},$$

The center frequency *f*_0_ can move to zero frequency in the spectrum of *z*(*t*) by shifting $e^{-j2\pi f_{0}t}$ to the left. By applying an ideal low-pass filter (LPF) to *z*(*t*) with a cutoff frequency *f_c_* < *f_0_*, signal *z*_*l**p*_(*t*) can contain only the component of interest, as follows:

(A3) $$z_{lp}\left(t\right)=\frac{A(t)}{2}e^{j\varphi (t)},$$

(A4) $$A\left(t\right)=2\;\left|z_{lp}(t)\right|,$$

(A5) $$\varphi \left(t\right)=\arctan\left(\frac{imag(z_{lp}\left(t\right))}{real(z_{lp}\left(t\right))}\right).$$

Here, the term ‘ideal low-pass filter’ refers to the theoretical demodulation step in the analytic formulation. In practice, this operation was implemented using a digital LPF as part of the VFCDM algorithm.

When the modulating frequency is not fixed but varies as a function of time, the signal x(t) can be expressed as follows:

(A6) $$x\left(t\right)=dc\left(t\right)+A\left(t\right)\left(\int_{0}^{t}cos(2\pi f\left(\tau \right)d\tau +\varphi (t))\right)$$

Similar to Equations (A1) and (A2), multiplying Equation (A6) by $e^{-j\int_{0}^{t}2\pi f\left(\tau \right)d\tau }$ produces both instantaneous amplitude, *A*(*t*), and instantaneous phase *φ*(*t*), as follows:

(A7) $$z\left(t\right)=x\left(t\right)e^{-j\int_{0}^{t}2\pi f\left(\tau \right)d\tau }=dc\left(t\right)e^{-j\int_{0}^{t}2\pi f\left(\tau \right)d\tau }+\frac{A(t)}{2}e^{j\varphi (t)}+\frac{A(t)}{2}e^{-j\int_{0}^{t}4\pi f\left(\tau \right)d\tau }$$

From Equation (A7), by applying an ideal LPF to z(t) with a cutoff frequency *f_c_* < *f_0_*, the filtered signal *z*_*l**p*_(*t*) is obtained with the same instantaneous amplitude A(t) and phase φ(t) as provided in Equations (A4) and (A5). The instantaneous frequency is given by

(A8) $$f\left(t\right)=f_{0}+\frac{1}{2\pi }\frac{d\varphi (t)}{dt}$$

Using a sampling frequency of 4 kHz, VFCDM decomposes the signals into spectral components centered at frequencies ranging from 80 to 1840 Hz, in 160 Hz increments, yielding a total of 12 components. For our analysis, we reconstructed the signal by summing components 2 through 7, corresponding to a frequency range of 160–1120 Hz. The first decomposition component (centered at 80 Hz) was excluded because it lies below the dominant SKNA band and overlaps with residual ECG PQRST activity and other low-frequency components not characteristic of SKNA.

The reconstructed signals were then normalized to unit variance and denoted as *X*′. The instantaneous amplitude of *X*′ was subsequently obtained using the Hilbert transform, as follows:

(A9) $$Y^{\prime }\left(t\right)=\frac{1}{\pi }p.v\int_{-\infty }^{\infty }\frac{X^{\prime }(\tau )}{t-\tau }d\tau$$

where p.v represents the Cauchy principal value. As *X*′(*t*) and *Y*′(*t*) form the complex conjugate pair, an analytic signal, *Z*(*t*), is defined as follows:

(A10) $$Z\left(t\right)=X^{\prime }\left(t\right)+iY^{\prime }\left(t\right)=a\left(t\right)e^{j\theta (t)}$$

(A11) $$a\left(t\right)={\left[{X^{\prime }}^{2}\left(t\right)+{Y^{\prime }}^{2}\left(t\right)\right]}^{1/2}$$

(A12) $$\theta \left(t\right)=arctan(\frac{Y^{\prime }\left(t\right)}{X^{\prime }\left(t\right)})$$

Finally, TVSKNA was calculated by applying a moving average filter with a 100 ms window to *a*(*t*) in Equation (A11).

## Appendix B

### Appendix B.1. Approximate Entropy (ApEn)

First, a time series *u*(*n*) consists of data points that are equally spaced in the time domain: *u*(1), *u*(2), … *u*(*N*), where *N* is the total number of data points. A sequence of vectors *x*(*i*) is then constructed for each 1≤i≤N−m+1, as follows–this process is known as embedding:

(A13) $$x\left(i\right)=\left[u\left(i\right),u\left(i+1\right),\ldots u\left(i+m-1\right)\right]$$

where *m*, a positive integer, represents the length of the data window.

The number of template vector pairs *x*(*i*), *x*(*j*), whose Chebyshev distance is smaller than a tolerance *r* (a positive real number), is defined as follows for 1≤i,j≤N−m+1:

(A14) $$C_{i}^{m}\left(r\right)=\frac{\left(number\;of\;j\;such\;that\;d[x\left(i\right),\;x\left(j\right)]\leq r\right)}{N-m+1}$$

The distance between two vectors *x*(*i*) and *x*(*j*) is defined as

(A15) $$d\left[x\left(i\right),x\left(j\right)\right]=\underset{k=1,2,\ldots m}{\max}\left(|x{\left(i\right)}_{k}-x{\left(j\right)}_{k}|\right)=\underset{k=1,2,\ldots m}{\max}\left(|u(i+k-1)-u(j+k-1)|\right)$$

In the case where *i* = *j*, *x*(*j*) is matched against itself, *x*(*i*), which is referred to as self-matching. Finally, ApEn is calculated as

(A16) $$ApEn\left(m,r,N\right)\left(u\right)=\emptyset^{m}\left(r\right)-\emptyset^{m+1}\left(r\right),$$

where $\emptyset^{m}\left(r\right)={\left(N-m+1\right)}^{-1}\sum_{i=1}^{N-m+1}logC_{i}^{m}(r)$ with 1≤i≤N−m+1.

Two parameters are specified: the embedding dimension (*m*) and the tolerance (*r*), which are a positive integer and a positive real number, respectively. The number of vector pairs to be compared denoted as *n*, is defined as N − *m* + 1.

### Appendix B.2. Sample Entropy (SampEn)

SampEn is calculated as follows:

(A17) $$SampEn\left(m,r,N\right)=\;-\ln\frac{A^{m}(r)}{B^{m}(r)},$$

where

$$A^{m}\left(r\right)=number\;of\;template\;vector\;pains\;with\;d\left[X_{m+1}\left(i\right),X_{m+1}\left(j\right)\right]<r,$$

(A18) $$B^{m}\left(r\right)=number\;of\;template\;vector\;pains\;with\;d\left[X_{m}\left(i\right),X_{m}\left(j\right)\right]<r,$$

The distance $d\left[x\left(i\right),x\left(j\right)\right]$ is typically defined same way as Equation (A15) but with *i ≠ j*. Therefore, SampEn does not have the self-matching problem. As $B^{m}\left(r\right)$ is always greater than $A^{m}\left(r\right)$, SampEn values are always zero or greater.
