# Supplementary material for: Complexity Analysis of Skin Nerve Activity for Quantitative Assessment of Acute Sympathetic Nervous System Activation
Source: Sensors (Basel). 2026 Mar 4;26(5):1611. doi: 10.3390/s26051611 (PMC12987309; doi:10.3390/s26051611)
Supplement: Supplementary file 1 [file sensors-26-01611-s001.zip › sensors-4161219-supplementary.pdf]

Supplementary Materials

Complexity Analysis of Skin Nerve Activity for Quantitative Assessment of Acute Sympathetic Nervous System Activation

Youngsun Kong[1]*, Yubin Choi[2], Farnoush Baghestani[1], Dong-Guk Shin[3], I-Ping Chen[2], and Ki Chon[1]

1 Department of Biomedical Engineering, University of Connecticut, Storrs, CT 06269, USA

2 Department of Endodontics, University of Connecticut Health, Farmington, CT 06032, USA

3 Department of Computer Science and Engineering, University of Connecticut, Storrs, CT 06269, USA

* Correspondence: youngsun.kong.phd@gmail.com

Table S1. Effect size (Cohen's *d*) for the experimental dataset (95% CI).

		VM	TG (NP vs. CSP-)	TG (NP vs. CSP+)	TG (CSP- vs. CSP+)
iSKNA	S.D.	3.033** (2.422, 3.637)	1.468** (0.952, 1.984)	2.047** (1.504, 2.591)	0.579* (0.225, 0.934)
	Mobility	-3.451** (-4.097, -2.796)	-1.488** (-2.004, -0.971)	-2.072** (-2.617, -1.527)	-0.584* (-0.939, -0.23)
	Complexity	2.98** (2.375, 3.576)	1.057** (0.555, 1.559)	1.458** (0.94, 1.975)	0.401 (0.05, 0.752)
	KFD	-2.831** (-3.411, -2.243)	-1.531** (-2.05, -1.013)	-2.098** (-2.645, -1.552)	-0.567* (-0.921, -0.213)
	SampEn	-3.461** (-4.109, -2.805)	-1.393** (-1.906, -0.88)	-2.02** (-2.563, -1.478)	-0.628* (-0.983, -0.272)
	ApEn	-3.46** (-4.108, -2.804)	-1.427** (-1.941, -0.913)	-2.065** (-2.61, -1.521)	-0.638* (-0.994, -0.282)
TVSKNA	S.D.	1.972** (1.463, 2.473)	1.638** (1.116, 2.161)	2.221** (1.668, 2.774)	0.583* (0.228, 0.937)
	Mobility	-2.866** (-3.449, -2.274)	-1.372** (-1.884, -0.86)	-2.048** (-2.591, -1.504)	-0.676** (-1.033, -0.319)
	Complexity	2.256** (1.723, 2.782)	1.012** (0.511, 1.513)	1.466** (0.948, 1.984)	0.454* (0.102, 0.806)
	KFD	-1.928** (-2.423, -1.425)	-1.227** (-1.735, -0.72)	-1.88** (-2.416, -1.345)	-0.653** (-1.009, -0.297)
	SampEn	-2.534** (-3.085, -1.975)	-1.381** (-1.894, -0.869)	-2.087** (-2.633, -1.541)	-0.706** (-1.063, -0.348)
	ApEn	-2.648** (-3.209, -2.078)	-1.417** (-1.931, -0.903)	-2.108** (-2.655, -1.561)	-0.691** (-1.048, -0.334)

Asterisks indicate statistical significance (* p<.05, ** p<.001; Tukey-adjusted).

Table S2. AUC for the experimental dataset (95% CI).

		VM	TG (NP vs. CSP-)	TG (NP vs. CSP+)	TG (CSP- vs. CSP+)
iSKNA	S.D.	0.979 (0.954, 1.0)	0.948 (0.883, 1.0)	0.953 (0.92, 0.986)	0.634 (0.51, 0.757)
	Mobility	0.956 (0.906, 1.0)	0.834 (0.708, 0.96)	0.853 (0.776, 0.93)	0.619 (0.495, 0.744)
	Complexity	0.958 (0.911, 1.0)	0.814 (0.682, 0.945)	0.834 (0.743, 0.925)	0.619 (0.495, 0.742)
	KFD	0.923 (0.836, 1.0)	0.918 (0.836, 1.0)	0.898 (0.844, 0.953)	0.564 (0.436, 0.692)
	SampEn	0.952 (0.899, 1.0)	0.823 (0.695, 0.95)	0.855 (0.78, 0.93)	0.607 (0.48, 0.735)
	ApEn	0.954 (0.9, 1.0)	0.83 (0.705, 0.954)	0.856 (0.78, 0.932)	0.617 (0.493, 0.741)
TVSKNA	S.D.	0.937 (0.879, 0.996)	0.993 (0.978, 1.0)	0.988 (0.974, 1.0)	0.631 (0.521, 0.741)
	Mobility	0.919 (0.839, 0.999)	0.83 (0.699, 0.96)	0.843 (0.763, 0.924)	0.625 (0.507, 0.744)
	Complexity	0.947 (0.891, 1.0)	0.825 (0.693, 0.957)	0.859 (0.773, 0.945)	0.606 (0.479, 0.733)
	KFD	0.871 (0.756, 0.987)	0.802 (0.659, 0.945)	0.858 (0.79, 0.926)	0.632 (0.5, 0.764)
	SampEn	0.863 (0.746, 0.98)	0.818 (0.684, 0.952)	0.854 (0.782, 0.925)	0.636 (0.508, 0.764)
	ApEn	0.883 (0.78, 0.986)	0.832 (0.702, 0.961)	0.856 (0.783, 0.93)	0.627 (0.503, 0.752)

Table S3. Repeated measures correlation coefficients for experimental dataset (95% CI).

	iSKNA	TVSKNA
S.D.	0.559 (0.428, 0.666)	0.601 (0.478, 0.7)
Mobility	-0.523 (-0.637, -0.386)	-0.544 (-0.654, -0.411)
Complexity	0.428 (0.276, 0.558)	0.415 (0.262, 0.548)
KFD	-0.496 (-0.615, -0.354)	-0.526 (-0.64, -0.389)
SampEn	-0.506 (-0.624, -0.367)	-0.544 (-0.655, -0.411)
ApEn	-0.523 (-0.638, -0.386)	-0.555 (-0.664, -0.424)

All indices showed statistical significance (p<.001; Tukey-adjusted).

Table S4. Effect size (Cohen's *d*) for clinical dataset (95% CI).

		NP vs. CSP-	NP vs. CSP+	CSP- vs. CSP+
iSKNA	S.D.	0.141 (-0.23,0.511)	1.009** (0.668,1.351)	0.869** (0.472,1.266)
	Mobility	-0.365 (-0.734,0.005)	-1.121** (-1.463,-0.779)	-0.756** (-1.147,-0.366)
	Complexity	0.322 (-0.047,0.691)	1.092** (0.75,1.434)	0.77** (0.379,1.161)
	KFD	-0.236 (-0.599,0.128)	-0.86** (-1.192,-0.529)	-0.625* (-1.006,-0.243)
	SampEn	-0.354 (-0.724,0.016)	-1.075** (-1.417,-0.733)	-0.721** (-1.112,-0.329)
	ApEn	-0.355 (-0.724,0.014)	-1.095** (-1.437,-0.753)	-0.74** (-1.131,-0.35)
TVSKNA	S.D.	0.273 (-0.095,0.64)	1.173** (0.83,1.516)	0.9** (0.508,1.292)
	Mobility	-0.461* (-0.835,-0.088)	-1.037** (-1.381,-0.694)	-0.576* (-0.969,-0.183)
	Complexity	0.315 (-0.056,0.686)	0.899** (0.56,1.237)	0.583* (0.192,0.975)
	KFD	-0.252 (-0.622,0.119)	-0.721** (-1.056,-0.386)	-0.469* (-0.859,-0.079)
	SampEn	-0.442 (-0.817,-0.068)	-0.981** (-1.324,-0.639)	-0.539* (-0.933,-0.145)
	ApEn	-0.459* (-0.833,-0.084)	-1.015** (-1.358,-0.672)	-0.557* (-0.95,-0.163)

Asterisks indicate statistical significance (* *p*<.05, ** *p*<.001; Tukey-adjusted).

Table S5. AUC values for non-severe-anxiety (NSA) and severe-anxiety (SA) groups from clinical dataset (95% CI).

		NP vs. CSP-		NP vs. CSP+		CSP- vs. CSP+	
		NSA	SA	NSA	SA	NSA	SA
iSKNA	S.D.	0.49 (0.38,0.59)	0.62 (0.38,0.86)	0.74 (0.66,0.81)	**0.82 (0.62,1.0)**	0.74 (0.65,0.84)	0.71 (0.48,0.94)
	Mobility	0.65 (0.54,0.75)	0.46 (0.21,0.71)	0.69 (0.61,0.77)	**0.87 (0.67,1.0)**	0.56 (0.46,0.67)	**0.81 (0.60,1.0)**
	Complexity	0.66 (0.55,0.76)	0.46 (0.21,0.71)	0.69 (0.60,0.77)	**0.86 (0.66,1.0)**	0.56 (0.45,0.66)	**0.82* (0.64,1.0)**
	KFD	0.65 (0.55,0.74)	0.51 (0.260.76)	0.64 (0.55,0.73)	**0.81 (0.61,1.0)**	0.53 (0.43,0.64)	**0.82* (0.63,1.0)**
	SampEn	0.64 (0.54,0.75)	0.48 (0.23,0.72)	0.68 (0.60,0.77)	**0.83 (0.62,1.0)**	0.56 (0.45,0.66)	**0.81* (0.60,1.0)**
	ApEn	0.65 (0.54,0.76)	0.49 (0.24,0.73)	0.69 (0.61,0.77)	**0.86 (0.66,1.0)**	0.56 (0.45,0.67)	**0.82* (0.61,1.0)**
TVSKNA	S.D.	0.53 (0.42,0.65)	0.55 (0.30,0.80)	0.75 (0.67,0.82)	**0.86 (0.67,1.0)**	0.71 (0.62,0.81)	**0.83 (0.64,1.0)**
	Mobility	0.66 (0.56,0.76)	0.43 (0.18,0.68)	0.67 (0.58,0.75)	**0.9* (0.75,1.0)**	0.51 (0.40,0.62)	**0.87** (0.73,1.0)**
	Complexity	0.66 (0.55,0.76)	0.53 (0.28,0.78)	0.65 (0.56,0.73)	**0.88* (0.71,1.0)**	0.49 (0.38,0.60)	0.76* (0.56,0.97)
	KFD	0.60 (0.49,0.71)	0.52 (0.25,0.79)	0.59 (0.50,0.68)	0.7 (0.44,0.96)	0.50 (0.39,0.61)	0.7 (0.44,0.96)
	SampEn	0.66 (0.55,0.76)	0.42 (0.17,0.67)	0.66 (0.57,0.74)	**0.96**(0.88,1.0)**	0.51 (0.40,0.62)	**0.91** (0.79,1.0)**
	ApEn	0.66 (0.55,0.76)	0.42 (0.17,0.67)	0.67 (0.58,0.75)	**0.91* (0.78,1.0)**	0.51 (0.41,0.62)	**0.89**(0.77,1.0)**

Asterisks indicate significant difference between NSA and SA groups (* *p*<.05, ** *p*<.001; Tukey-adjusted). Bold fonts indicate AUC values ≥ 0.80.

Table S6. Repeated measures correlation coefficients for clinical dataset (95% CI).

	iSKNA		TVSKNA	
	NSA	SA	NSA	SA
S.D.	0.501** (0.375, 0.609)	0.658** (0.378, 0.828)	0.494** (0.367, 0.604)	0.704** (0.448, 0.853)
Mobility	-0.502** (-0.610, -0.375)	-0.668** (-0.833, -0.392)	-0.463** (-0.577, -0.331)	-0.706** (-0.854, -0.452)
Complexity	0.495** (0.368, 0.604)	0.674** (0.401, 0.836)	0.432** (0.296, 0.551)	0.547* (0.219, 0.764)
KFD	-0.437** (-0.555, -0.302)	-0.509* (-0.741, -0.167)	-0.378** (-0.504, -0.236)	-0.536* (-0.757, -0.203)
SampEn	-0.484** (-0.595, -0.355)	-0.667** (-0.833, -0.391)	-0.439** (-0.557, -0.304)	-0.721** (-0.862, -0.475)
ApEn	-0.495** (-0.604, -0.368)	-0.665** (-0.832, -0.389)	-0.453** (-0.569, -0.320)	-0.710** (-0.856, -0.458)

Asterisks indicate statistical significance (* *p*<.05, ** *p*<.001; Tukey-adjusted). NSA: non-severe anxiety, SA: severe anxiety.

Table S7. Cohen's *d* (95% CI) for additional entropy and complexity measures.

	iSKNA			TVSKNA		
	VM	CSP+ (TG)	CSP+ (Dental)	VM	CSP+ (TG)	CSP+ (Dental)
DFA	1.47**<br>(1, 1.94)	-1.68**<br>(-2.21, -1.16)	-0.35<br>(-0.68, -0.02)	1.15**<br>(0.70–1.6)	-1.27<br>(-1.78, -0.75)	-0.4*<br>(-0.73, -0.07)
HFD	-0.03<br>(-0.44, 0.38)	0.96**<br>(0.46, 1.47)	0.35<br>(0.01, 0.68)	1.14**<br>(0.69, 1.58)	-0.52<br>(-1.01,-0.02)	-0.28<br>(-0.61, 0.05)
PFD	-0.23<br>(-0.65, 0.19)	0.60*<br>(0.11, 1.1)	0.17<br>(-0.16, 0.5)	0.19<br>(-0.22, 0.61)	-1*<br>(-1.51, -0.50)	-0.47*<br>(-0.80, -0.14)
PermEn	-0.23<br>(-0.64, 0.19)	0.61*<br>(0.12, 1.11)	0.18<br>(-0.15, 0.51)	0.19<br>(-0.23, 0.6)	-1*<br>(-1.5, -0.5)	-0.48*<br>(-0.81, -0.14)
SpectlEn	-4.01**<br>(-4.73, -3.29)	0.43<br>(-0.07, 0.92)	1.18**<br>(0.84, 1.52)	-3.38**<br>(-4.02, -2.73)	1.16**<br>(0.65, 1.66)	1.18**<br>(0.83, 1.52)
SVDEn	-0.32<br>(-0.73,0.09)	-1.1*<br>(-1.6, -0.59)	-0.19<br>(-0.51, 0.13)	-0.81**<br>(-1.23, -0.38)	-0.94<br>(-1.44, -0.44)	-0.10<br>(-0.44, 0.23)

Statistical analyses were performed using linear mixed-effects models (LMER) with Participant as a random intercept. Pairwise comparisons were obtained using estimated marginal means (emmeans) with Tukey adjustment. For Higuchi fractal dimension, the maximum window length was set to $k = 10$. Asterisks indicate statistical significance ($p < .05$, ** $p < .001$; Tukey-adjusted). VM: Valsalva Maneuver, CSP: clinically significant pain, TG: thermal grill, DFA: Detrended Fluctuation Analysis, HFD: Higuchi Fractal Dimension, PFD: Petrosian Fractal Dimension, PermEn: Permutation Entropy, SpectlEn: Spectral Entropy, SVDEn: Singular Value Decomposition Entropy.

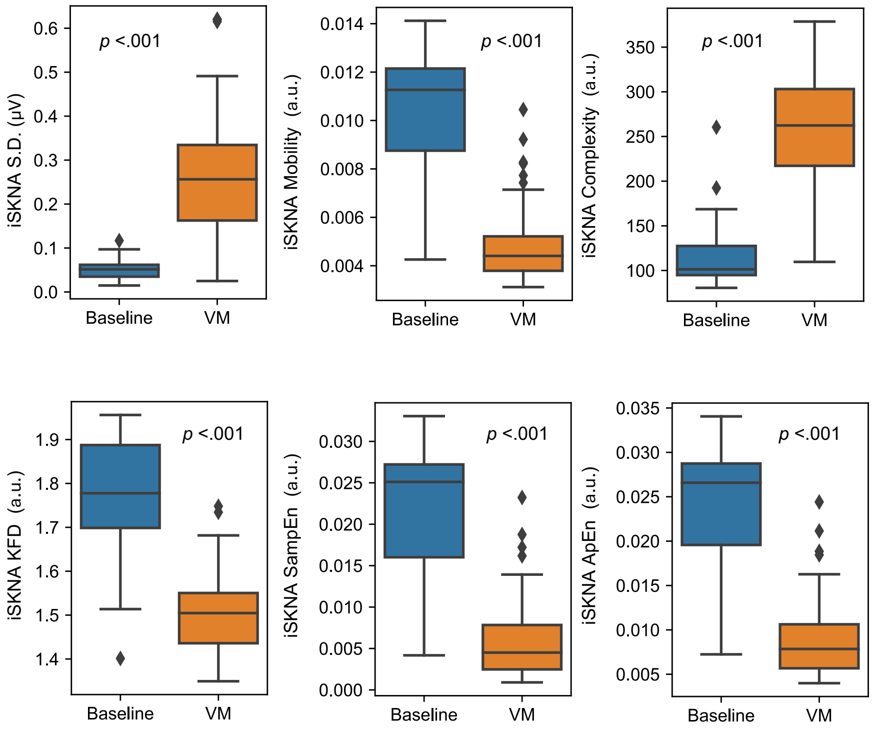

(a)

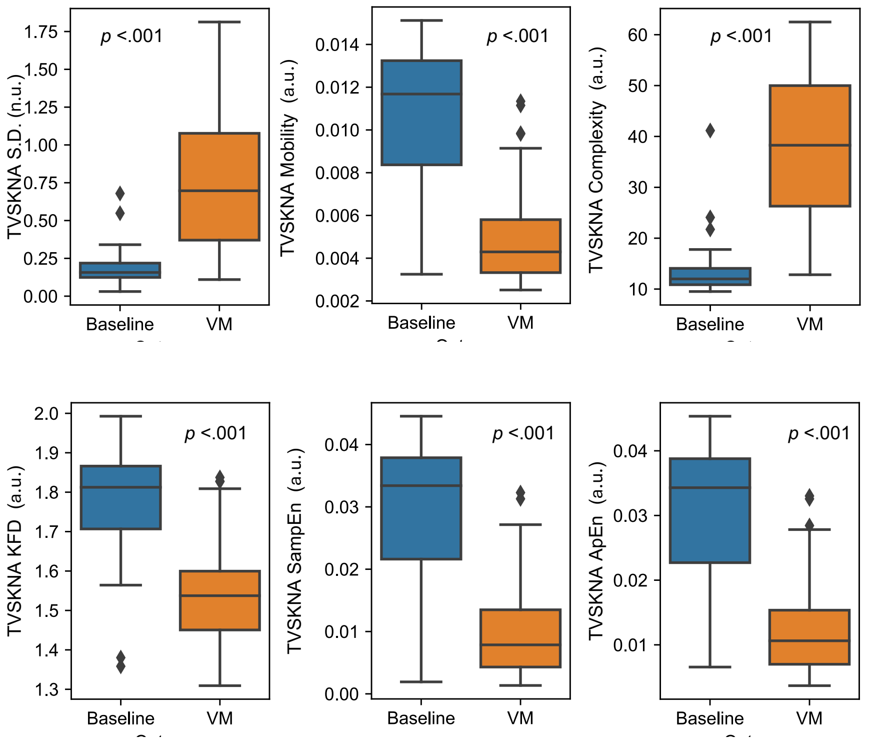

(b)

Figure S1. Boxplots for (a) iSKNA and (b) TVSKNA during VM.

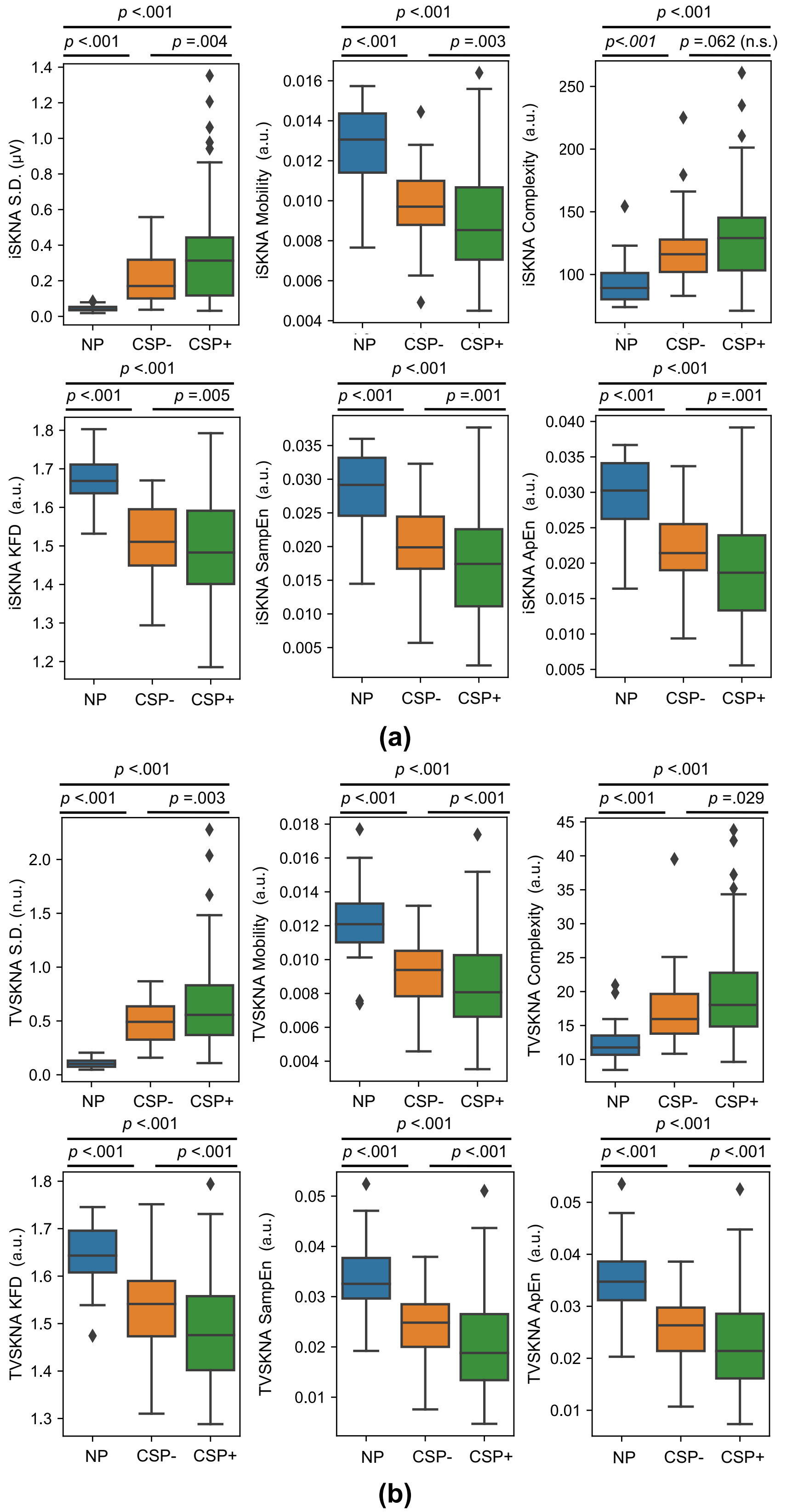

Figure S2. Boxplots for (a) iSKNA and (b) TVSKNA during thermal grill (TG) pain

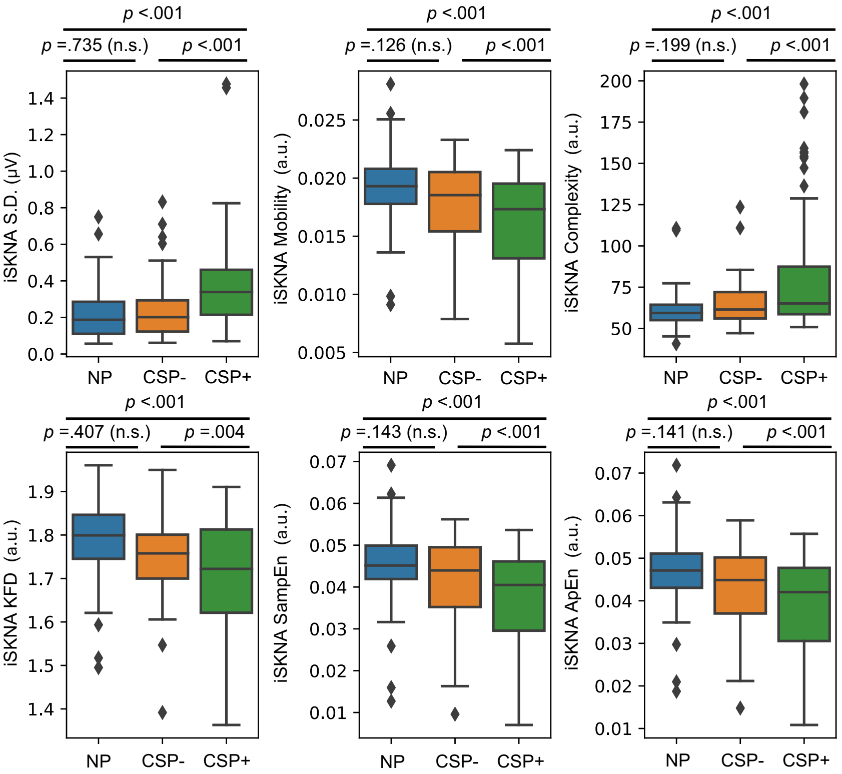

(a)

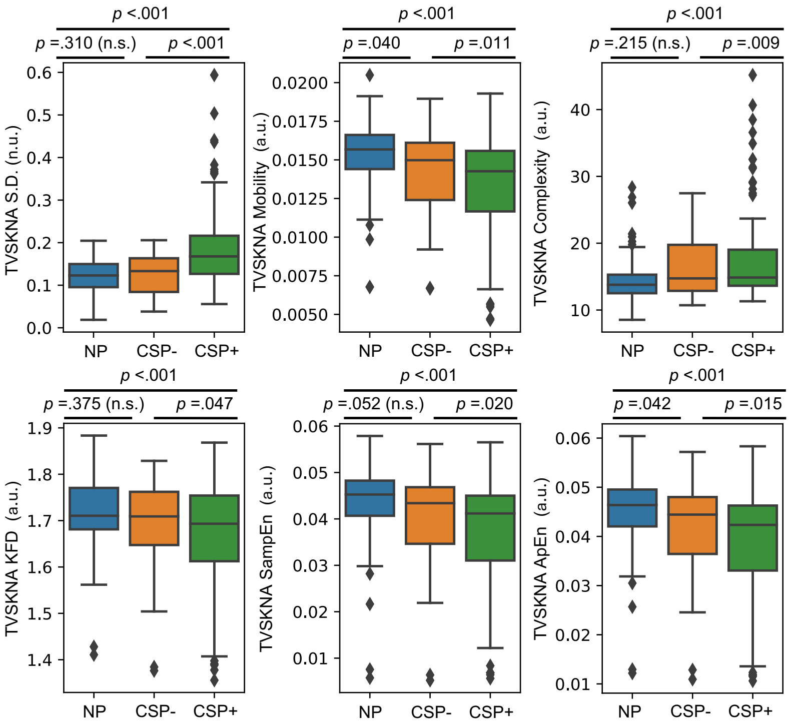

(b)

Figure S3. Boxplots for (a) iSKNA and (b) TVSKNA during cold test

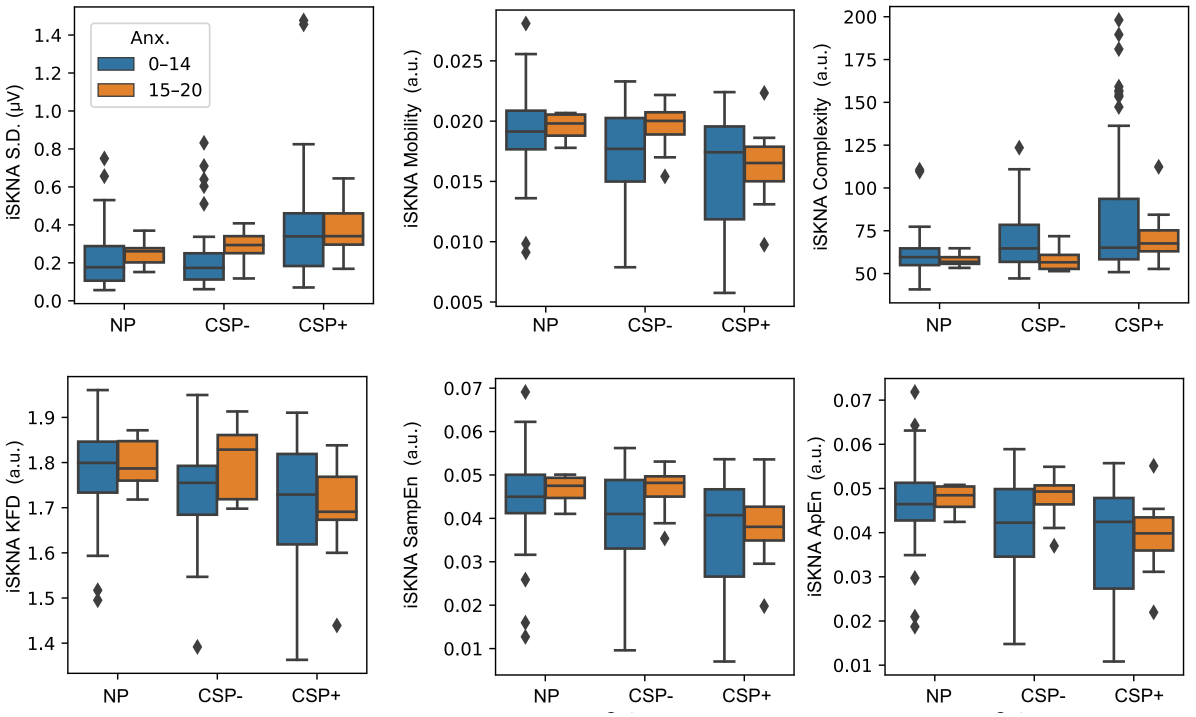

(a)

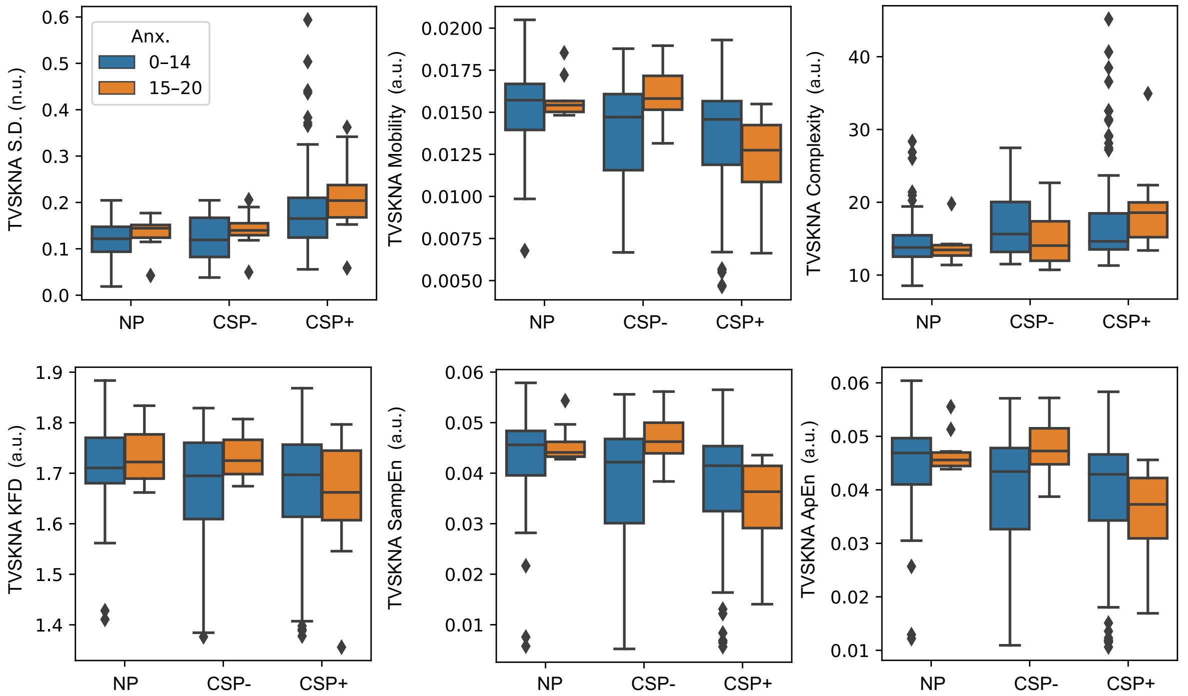

(b)

Figure S4. Boxplots for iSKNA (a) and TVSKNA (b) between non-severe and severe anxiety groups from clinical dataset. None of indices exhibited significant difference between those two groups. However, the severe anxiety group showed greater discriminatory between CSP+ and the other levels (See Table S5).

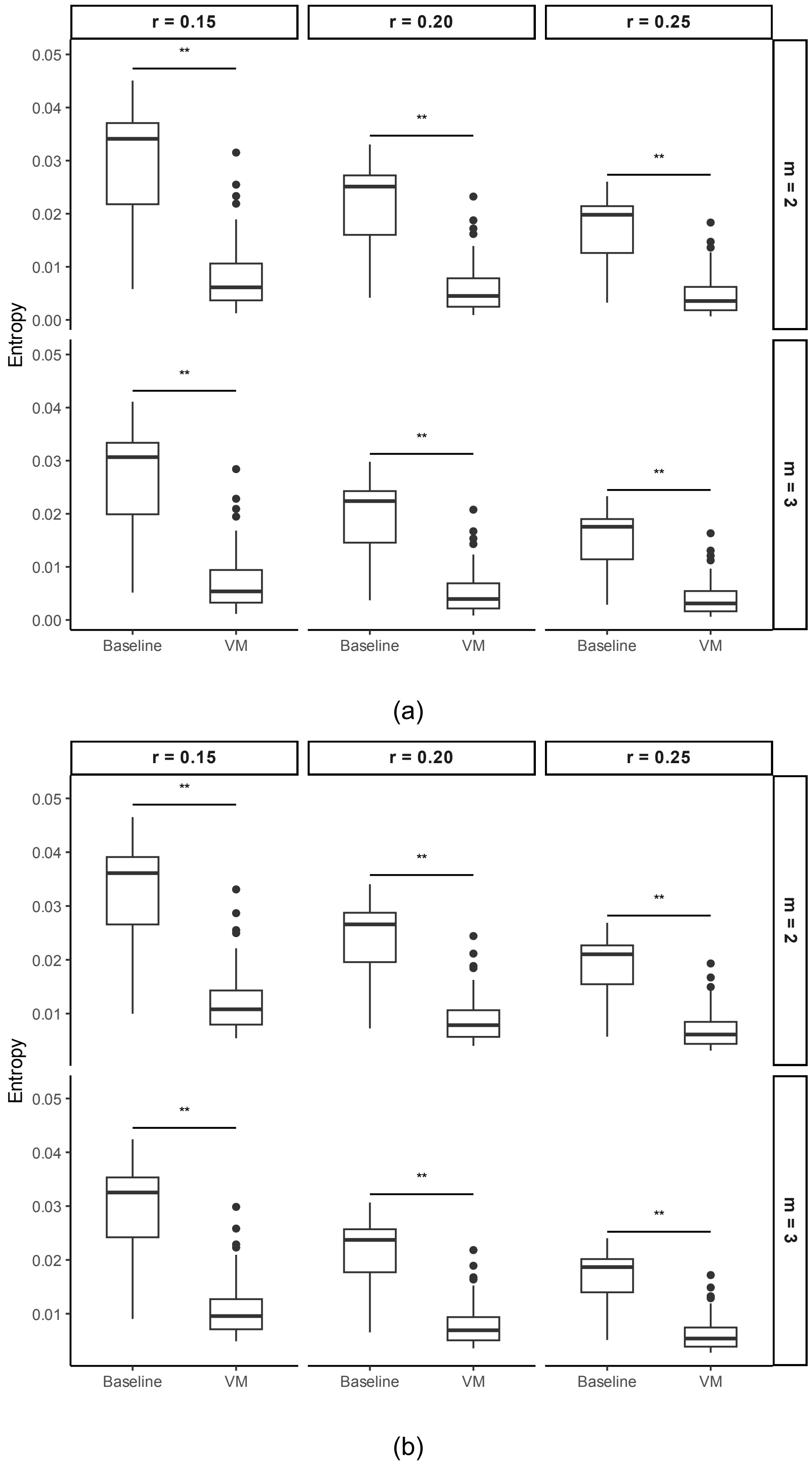

Figure S5. Parameter sensitivity of entropy-based discrimination from iSKNA during the Valsalva maneuver. (a) Sample entropy and (b) Approximate entropy. * p<.05, ** p<.001 (FDR-adjusted).

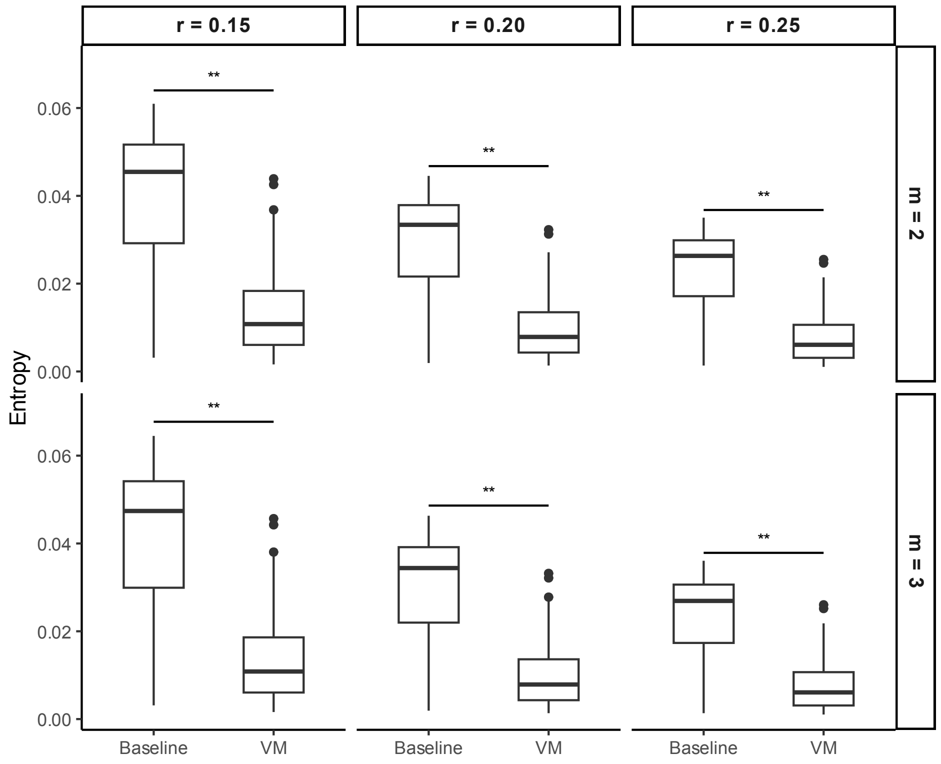

(a)

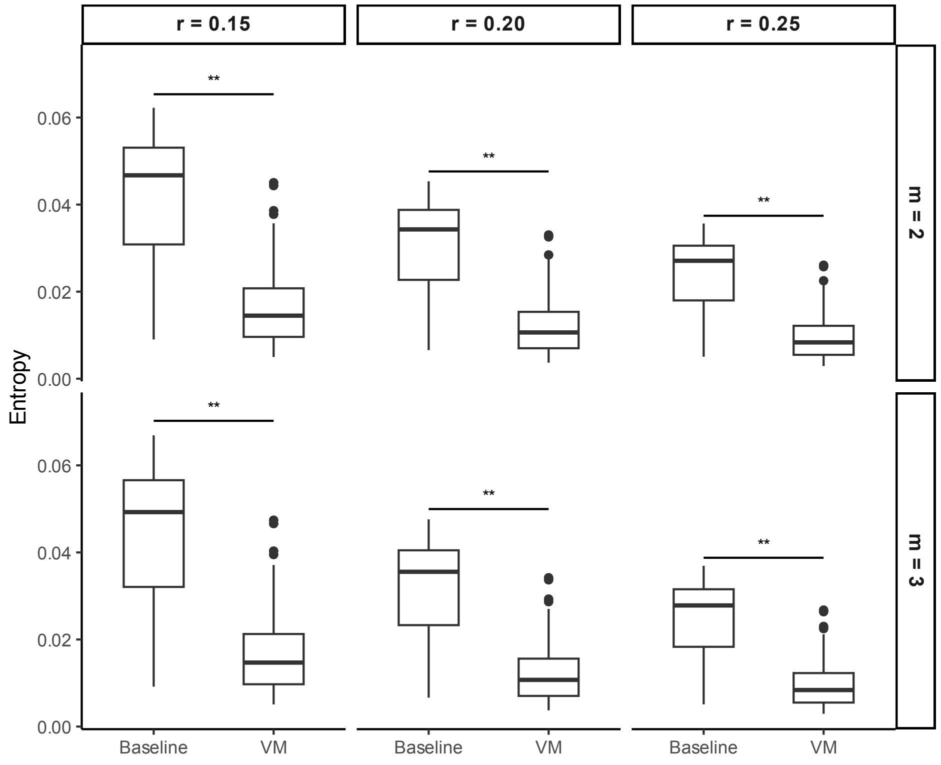

(b)

Figure S6. Parameter sensitivity of entropy-based discrimination from TVSKNA during the Valsalva maneuver. (a) Sample entropy and (b) Approximate entropy. * p<.05, ** p<.001 (FDR-adjusted).

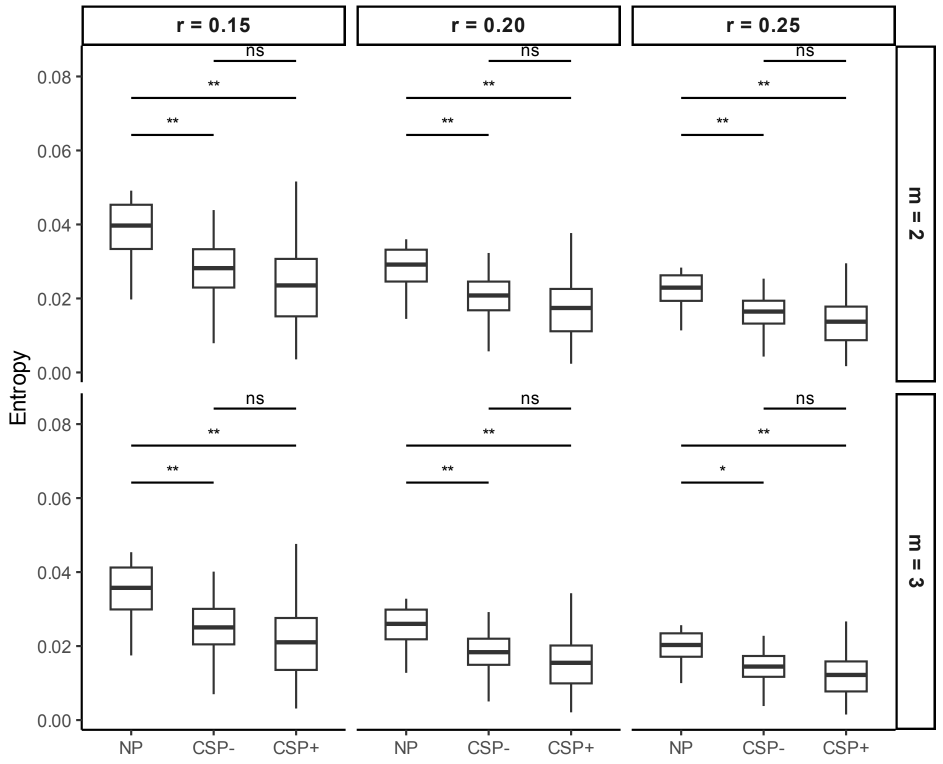

(a)

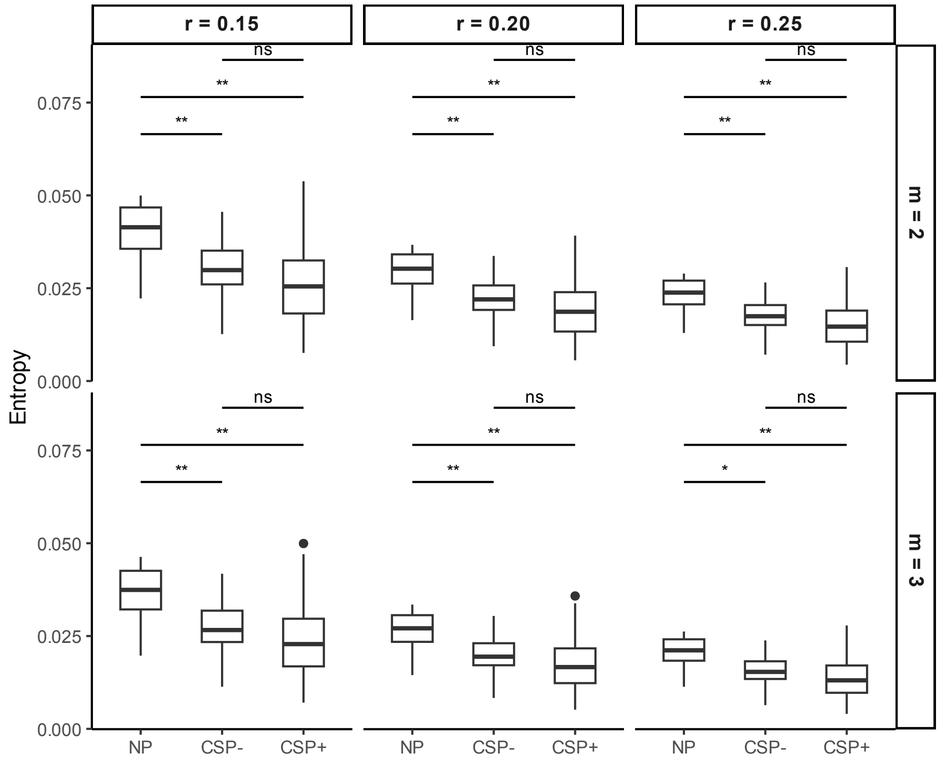

(b)

Figure S7. Parameter sensitivity of entropy-based discrimination from iSKNA during the thermal grill stimulation. (a) Sample entropy and (b) Approximate entropy. * p<.05, ** p<.001 (FDR-adjusted).

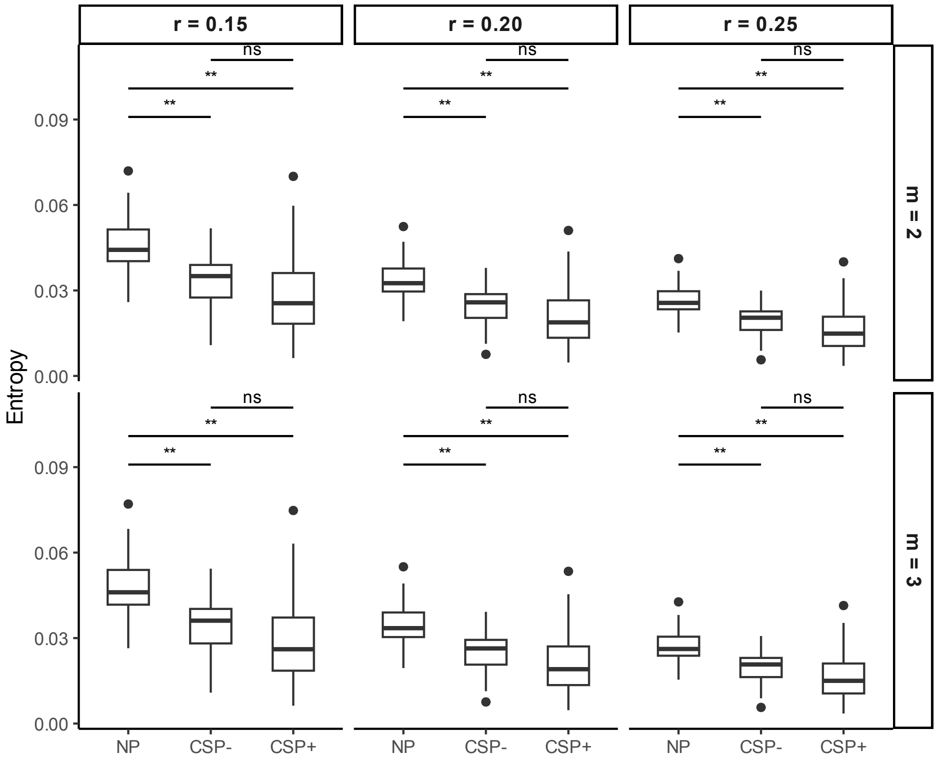

(a)

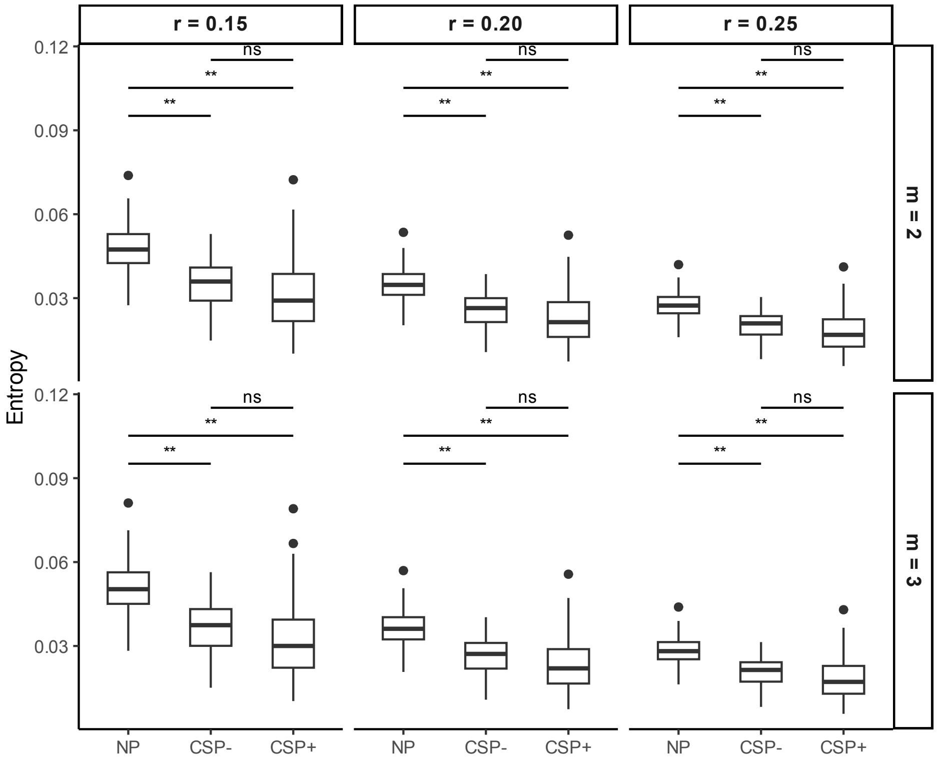

(b)

Figure S8. Parameter sensitivity of entropy-based discrimination from TVSKNA during the thermal grill stimulation. (a) Sample entropy and (b) Approximate entropy. * $p<.05$, ** $p<.001$ (FDR-adjusted).

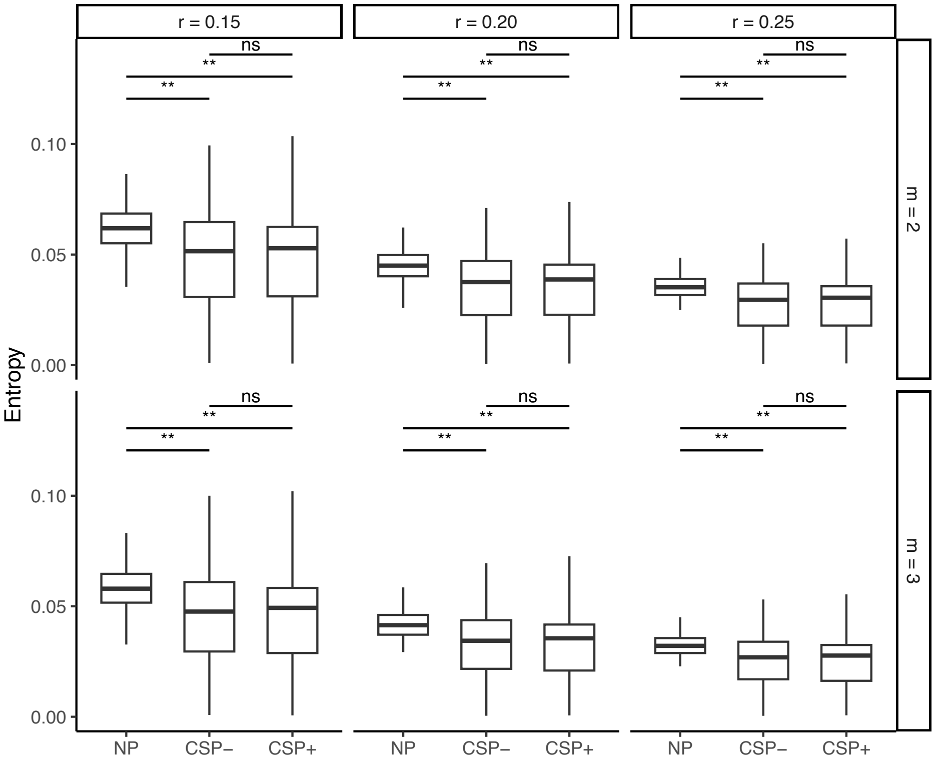

(a)

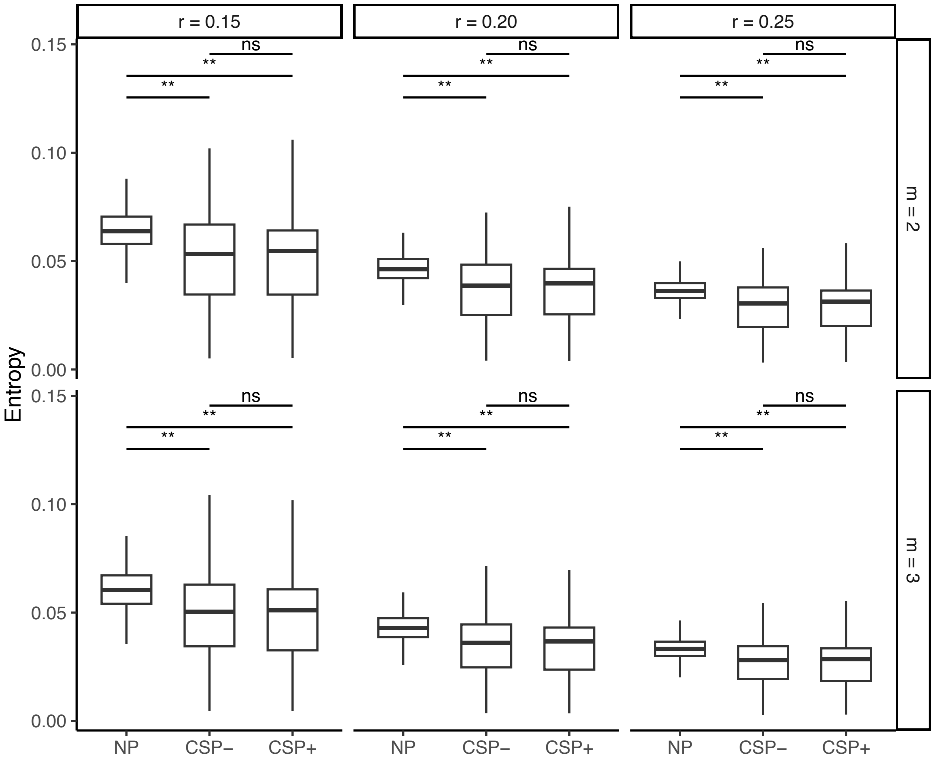

(b)

Figure S9. Parameter sensitivity of entropy-based discrimination from iSKNA during cold test. (a) Sample entropy and (b) Approximate entropy. * p<.05, ** p<.001 (FDR-adjusted).

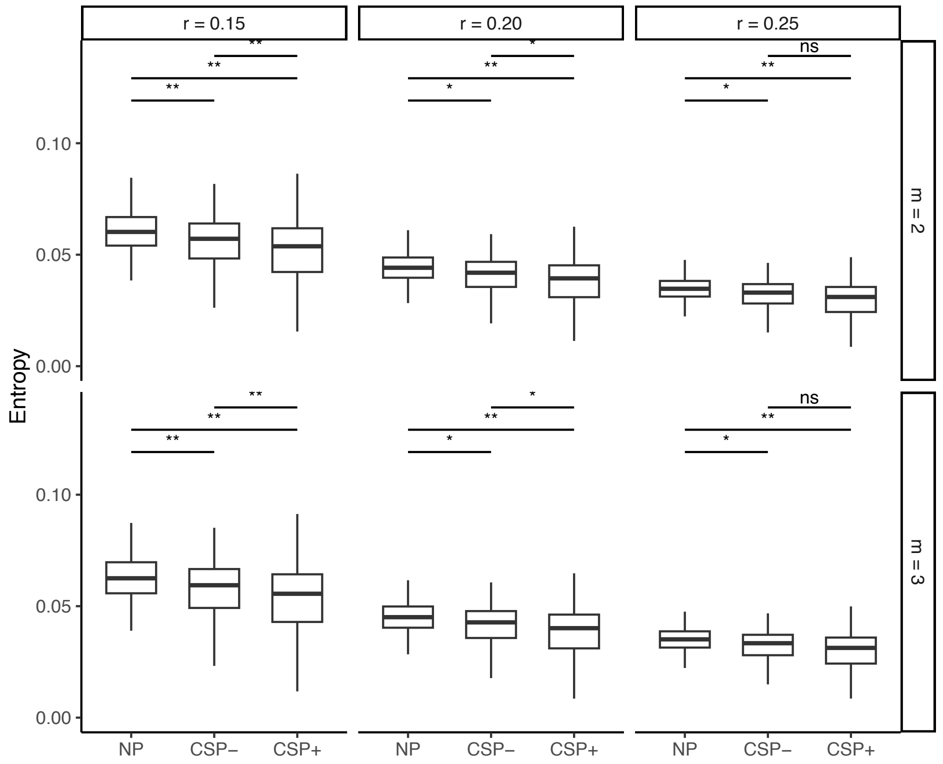

(a)

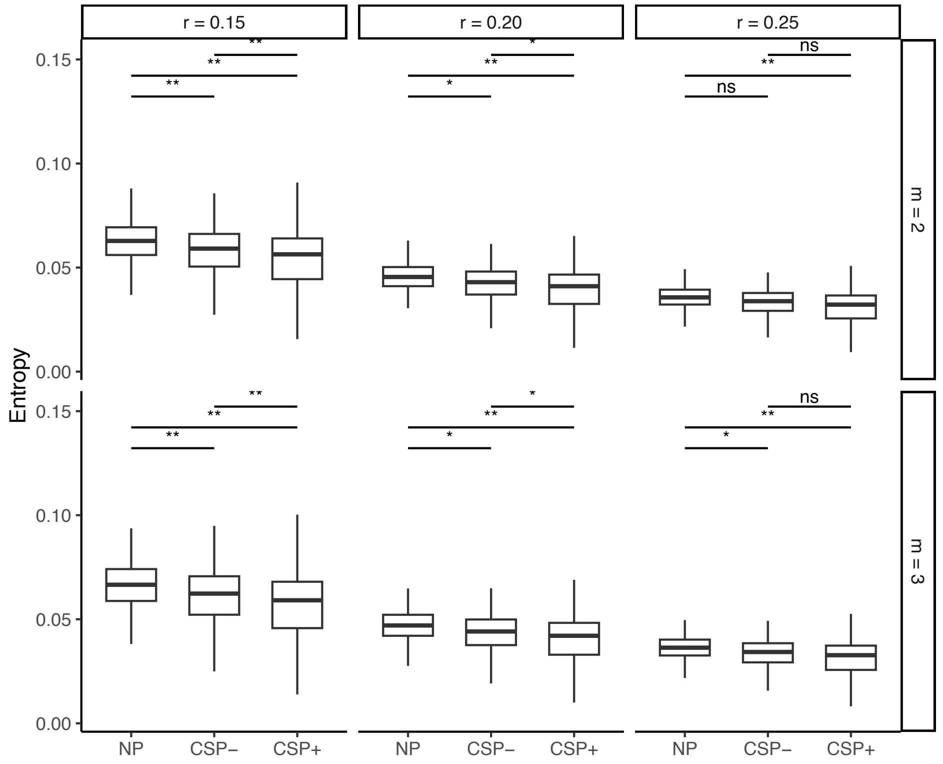

(b)

Figure S10. Parameter sensitivity of entropy-based discrimination from TVSKNA during cold test. (a) Sample entropy and (b) Approximate entropy. * p<.05, ** p<.001 (FDR-adjusted).
